# The scuttle flies (Diptera: Phoridae) of Iran with the description of *Mahabadphora aesthesphora* as a new genus and species

**DOI:** 10.1371/journal.pone.0257899

**Published:** 2021-10-13

**Authors:** Roya Namaki-Khameneh, Samad Khaghaninia, R. Henry L. Disney, Naseh Maleki-Ravasan

**Affiliations:** 1 Faculty of Agriculture, Department of Plant Protection, University of Tabriz, Tabriz, Iran; 2 Department of Zoology, University of Cambridge, Cambridge, United Kingdom; 3 Department of Parasitology, Pasteur Institute of Iran, Tehran, Iran; Nanjing Agricultural University, CHINA

## Abstract

Scuttle flies (Diptera: Phoridae) are mega-diverse and often synanthropic insects that play superb roles in various ecosystems. Identification of this group of insects is challenging due to their small size, morphological identification difficulties, niche diversity, and lack of taxonomic keys. To pave the way, an in-depth investigation was directed toward the scuttle flies in Iran using morphological and molecular data. A dichotomous key was also developed to identify the genus and species of the phorids reported in the country. The faunistic findings revealed the presence of about 22,000 (13,903 male and 8,097 female) phorid materials organized into 11 genera. *Megaselia* species (n = 13768), made up about 99% of the specimens studied. Moreover, 71 morphologically defined species belonging to nine genera were molecularly characterized using *COI*, *28S rRNA*, and *Arginine kinase* datasets. Excluding four *Megaselia* Rondani, 1856 species, our results specified that morphologically delimited species were in agreement with the molecular analyses inferred from the *COI*/*28S rRNA* and *COI*/*Arginine kinase* sequences with genetic distances and phylogenetic trees. According to the results of the present study and previously published data, the Phoridae recorded for Iran are a total of 97 species that are ordered in 13 genera and three subfamilies, including Chonocephalinae, Metopininae and Phorinae. By comparing the known world phorid genera, a new monotypic genus of scuttle flies, *Mahabadphora aesthesphora* gen. nov., sp. nov., was identified based on its morphological and molecular characteristics and included in an updated key. Our results could comprehensively determine the taxonomic status of scuttle flies in Iran, scrutinize their phylogenetic structures and facilitate their identification.

## Introduction

Scuttle flies (Diptera: Phoridae) are considered as one of the most abundant and diverse families of flies [1]. The adults resembling fruit flies can simply be recognized through the morphological (hump-backed outwards and reduced wing venations) and behavioral (escaping across a surface rather than flying) characteristics [2] These flies exploit a wide range of habitats and display miscellaneous feeding patterns from polyphagous saprophages and fungivorous to specialized predators and true parasites or parasitoids [3,4].

Larvae of many species are scavengers on decaying plant and animal organic matters, which are lively attractants to the female insects [5]. Other species are generally malacophagous [6] or parasitic on spiders [7], millipedes [8], and many insect orders, including Lepidoptera, Coleoptera, Hymenoptera, Isoptera etc. [9–12]. They are prominent parasitoids of particular arthropods, as well. For instance, *Pseudacteon* spp. are mainly acknowledged as promising biocontrol agents of *Solenopsis invicta* Buren, 1972, a very aggressive medically important ant [13]. Likewise, *Apocephalus paraponerae* Borgmeier, 1958 is a parasitoid of the giant tropical ant, *Paraponera clavata* (Fabricius, 1775) [14]. The occurrence of the *Megaselia scalaris* Loew, 1866, as a parasitoid of acarine tick *Boophilus microplus* (Canestrini, 1888), is also documented [15,16].

A few phorid species are significant agents of human facultative myiasis. *Megaselia scalaris* Loew, 1866 and *M*. *spiracularis* Schmitz, 1938 are examples of such species that are the causative agents of wound, ophthalmic, pneumonic, nasal, intestinal, urogenital, and nosocomial myiasis, worldwide [17–25].

Several species might provide valuable entomological facts in crime scene inquiries, particularly for post-mortem interval (PMI) estimations [26]. Due to the minute size, species such as *M*. *scalaris* can move to every enclosed and concealed environment and may possibly offer more accurate PMI assessments than calliphorid species [27]. The *Conicera tibialis* Schmitz, 1925, known as coffin fly, raises numerous generations in hidden settings, such as coffins or in shallow graves of buried remains [28]. Unlike two mentioned species, *Dohrniphora cornuta* (Bigot, 1857), a globally distributed species, often flies indoors and to burial settings [29]. Besides, the phorid flies can proficiently infest every kind of invertebrate and small vertebrate cultures [30–33]. Therefore, scuttle flies are recognized as insects of environmental, agricultural, medical and forensic significance. However, the detailed life history of most species is still far from entire perception and requires further surveys.

The Phoridae family has currently been classified into four subfamilies (Sciadocerinae, Chonocephalinae, Metopininae, and Phorinae), 260 genera, more than 4000 described species, and many more remain to be described [34,35]. In spite of being one of the largest families of insects in terms of number of know species, Phoridae has much less been studied in comparison with others due to their relatively small size and morphological identification difficulties. DNA profiling clearly arises as the most influential and reliable method for identifying insect individuals in divers taxonomic groups [36–41]. Mito-nuclear molecular markers have chiefly been utilized for the identification of phorid flies with forensic and medical superiorities [25,42]. Nonetheless, inadequate molecular surveys have sometimes been conducted to resolve taxonomic problems in other sets of scuttle flies. For example, mitochondrial and ribosomal RNA genes have been used for phylogenetic analysis of critical genera of Phoridae and related families [43]. Combinations of mitochondrial and nuclear genes have been engaged to assess the monophyly of *Anevrina* Lioy, 1864 and *Dohrniphora* Dahl, 1898 genera in distinct studies [44,45]. The *COI* and *28S rRNA* markers have been utilized to organize *Megaselia*, the most species-rich genus of scuttle flies, at the subgenus level [46]. The mito-nuclear markers of *COI*/*wingless* genes have also been utilized to discriminate body size biotypes as well as cryptic species of *Pseudacteon* Coquillett, 1907 [47,48].

The Phorid fauna of Iran has not practically been studied in many areas, and very limited studies have been conducted to identify species with medical [24] and agricultural [49–51] significance. The main objective of the current study was to offer a comprehensive review of the scuttle flies in Iran. This goal was achieved by applying an in-depth survey with the aid of combining morphology with molecular data, to identify and validate the scuttle fly species in the country. A morphological identification key of the scuttle flies in the country was developed, as well. Based on the findings, we could detect a new monotypic genus in West Azerbaijan Province relying on its morphological and molecular characteristics.

## Materials and methods

### Ethics statement

All experiments were performed in accordance with international and institutional ethics guidelines. No specific permissions were required for this study. The study did not involve endangered or protected species.

### Study area, sample collections, and morphological studies

Adult insects were captured in a malaise trap and by a standard insect net from grasslands and wetlands in Ardabil, East Azerbaijan, and West Azerbaijan Provinces (Iran), from May to September 2012–2018. Specimens were promptly killed in a potassium cyanide jar and kept in 70% ethanol at 4°C until analysis. Taxonomic sorting of trapped insects and initial identification of phorid flies were accomplished in the Entomology Laboratory of Tabriz University (East Azerbaijan Province). Detailed identification of the flies was carried out by preparing and mounting the specimen slides in Berlese’s medium in the Department of Zoology, University of Cambridge (UK), according to the Disney [52]. The slides were labeled, and pictures from the whole body of each fly were taken using a Nikon SMZ800N stereomicroscope equipped with a Nikon D5200 digital camera. The discriminative features of individual specimen were photographed by the Image Pro-Insight system attached to a compound microscope. The type materials of the new taxa were deposited in the University of Cambridge Museum of Zoology (UCMZ). Duplicate paratypes were used in molecular surveys or were preserved in the Insect Collection of Professor Hasan Maleki Milani (ICHMM), Tabriz, Iran.

### Molecular surveys

#### DNA extraction, PCR amplification, and sequencing

A total of 71 paratypes were subjected for molecular characterizations. Before DNA isolation, adult flies were separately washed thoroughly with 70% ethanol and centrifugation. Total genomic DNA was extracted from the whole body of each fly, using the GeneAll Exgene^TM^ Cell SV Mini Kit (Seoul, Korea) and according to the protocol for animal tissues. DNA concentration was evaluated by measuring the absorbance at 260 nm using a PowerWave XS Microplate Spectrophotometer (BioTek, Vermont, USA). DNA specimens were stored at -20°C until investigation.

Portions of three different loci, including one mitochondrial cytochrome oxidase I (*COI*; 831 bp) and two nuclear expansion segment D7 of *28S rRNA* (*28S*; 607 bp) and *Arginine kinase* (*AK*; 756 bp) genes, were amplified and sequenced using the oligonucleotide primers and thermal profiles specified in Table 1. All PCR amplifications were performed in a 25 μL volume using the *Taq* DNA Polymerase 2× Master Mix RED from Ampliqon (Denmark), with the subsequent mixtures: 1–2 μL of DNA extract (~0.1 μg), 12.5 μL of Master mix, 1 μL of each primer (10 mM), and 8.5–9.5 μL of sterile water. PCR products were checked via 1.5% agarose gel electrophoresis, followed by GreenViewer staining and photographing using a UV transilluminator. Fruitful amplicons were purified and sequenced at both directions by Genomin Company, Tehran, Iran.

**Table 1:** Details of the primer sequences and thermal profile used for the amplification of target genes.

Locus	Gene & Primer code	Sequence (5’ - 3’)	Thermal conditions	Product size (bp)	Reference
mitochondrial	*COI*-F (C1-j-2183) *COI*-R (TL2-n-3014)	CAACATTTATTTTGATTTTTTGG CCATTGCATCTGCCATATTA	D: 94°C for 30 sec; A: 47(52°C for 40 sec; E: 72°C for 1 min, 5 cycles 47°C, 35 cycles 52°C	∼830	[53]
nuclear	*28S*-F (Rc28p) *28S*-R (28E)	TGGTATGCGTAGAAGTGTTTGGC CCTTATCCCGAAGTTACG	D: 94°C for 1 min; A: 53°C for 1 min; E:72°C for 2 min, 30 cycles	∼600	[54]
	*AK*-F (AK183F) *AK*-R (AK939R)	GATTCTGGAGTCGGNATYTAYGCNCCYGAYGC GCCNCCYTCRGCYTCRGTGTGYTG	D: 94°C for 30 sec; A: 53°C for 30 sec; E:72°C for 1 min, 35 cycles	∼750	[55]

**Abbreviations**: *COI*: *cytochrome oxidase I*; *28S*: *28S rRNA*; *AK*: *Arginine kinase*; F: Forward; R: Reverse; bp: base pairs; D: denaturation; A: annealing; E: elongation.

#### Sequence and phylogenetic relationships analysis

The sequences obtained in this study (n = 143) and those retrieved from GenBank (n = 219 and n = 204) were respectively used to study genetic diversity in a single mode and also to reconstruct phylogenetic trees in the combined modes (S1 Table). The quality of raw sequences obtained herein was proofread using the Chromas 2.6.6 (Technelysium Pty Ltd., South Brisbane, Australia). The BLAST (Basic Local Alignment Search Tool) search was employed to compare under-investigated sequences. Multiple sequence alignments were conducted by Clustal Omega software [56]. The basic sequence statistics, including polymorphic and parsimony-informative sites, were analyzed by the aid of MEGA X software [57]. Interspecific and inter/intrageneric divergences for the studied gene sets were estimated with the suite of molecular genetic programs embedded in MEGA X using the Kimura two-parameter (K2P) distance model [58]. The combination of *COI*-*28S* and *COI*-*AK* gene sets was exploited to infer relationships. Phylogenetic relationships were examined using maximum likelihood (ML) and neighbour joining (NJ) algorithms with K2P correction models embedded in MEGA X software. Confidence of internal nodes was assessed by bootstrap analysis with 1,000 replicates. Sequences of the target genes in brachyceran fly species *Drosophila melanogaster* Meigen, 1830, *Glossina morsitans* Westwood, 1851 and *Musca domestica* Linnaeus, 1758 were designated as outgroups. All sequences achieved in this study were deposited in the GenBank database (S1 Table).

#### Literature review and providing an identification key

An extensive literature review was conducted based on a search of online scientific databases (Scientific Information Database, PubMed and Google Scholar) to find published reports on phorid flies in Iran before 30^th^ June 2020. Searches were performed in titles, abstracts, keywords, and full texts. Keywords for the search were Phorids AND fauna AND Iran, Iran AND Phoridae, and Iran AND scuttle flies. An updated dichotomous key to all known species of Phoridae in Iran, including specimens from the current study, was subsequently generated.

### Nomenclatural acts

The electronic edition of this paper follows to the necessities of the adjusted International Code of Zoological Nomenclature, and therefore the new names included herein are accessible under that Code from the electronic edition of this article. This published paper and the nomenclatural acts it comprises have been registered in ZooBank, the online registration organization for the ICZN. The ZooBank LSIDs (Life Science Identifiers) can be resolved and the associated information viewed through any standard web browser by appending the LSID to the prefix “http://zoobank.org/”. The LSID for this publication is: urn:lsid:zoobank.org:pub:9145941B-10BF-4B27-8B4C-D90006A857B5. The LSIDs of the all publications and species mentioned in the present project are available in the supplementary materials (S2 Table). The electronic edition of this work was published in a journal with an ISSN, and has been archived and is available from the following digital repositories: PubMed Central, LOCKSS.

## Results

### Morphological identifications

A total of 22,000 phorid materials, comprising of 13,903 males and 8,097 females, were collected during excursions within six consecutive years. A major part of the faunistic results of the sampled flies in the form of new taxa and records were published in reputable journals [59–64]. The remaining 2,250 specimens, including three genera (*Megaselia* Rondani, 1856, *Metopina* Macquart, 1835 and an unknown genus) and 19 species are herein reported (Table 2). The recent flies were gathered from six locations in East (Chichekli, Shanejan, Sharafkhaneh, and Sufiyan) and West (Khoy and Mahabad) Azerbaijan Provinces. *Megaselia* spp. included *M*. *angelicae* Wood, 1910; *M*. *berndseni* (Schmitz, 1919); *M*. *communiformis* (Schmitz, 1918); *M*. *hirtiventris* (Wood, 1909); *M*. *largifrontalis* Schmitz, 1939; *M*. *longipalpis* (Wood, 1910); *M*. *pallidizona* (Lundbeck, 1920); *M*. *perdistans* (Schmitz, 1924); *M*. *propinqua* (Wood, 1909); *M*. *pusilla* (Meigen, 1830); *M*. *ruficornis* (Meigen, 1830); *M*. *sandhui* Disney, 1981; *M*. *stigmatica* (Schmitz, 1920); *M*. *subpleuralis* (Wood, 1909); *M*. *spinicincta* (Wood, 1910); *M*. *tarsalis* (Wood, 1910) and *M*. *verna* Schmitz, 1932. A species of *Metopina perpusilla* (Six, 1878) was found, as well. To identify the unknown genus/species, the entire known world genera of Phoridae deposited in UCMZ was directly examined.

**Table 2:** List of taxa analyzed in this study, with their collection data, and hierarchical classification, as well as synopsis of their life history.

No.	Subfamily/Species	N (male-female)	Locality	Latitude	Longitude	Altitude (m)	Reference	Life history perspectives
	**Chonocephalinae**							
1	*Chonocephalus heymonsi* Strobbe, 1913	---	Fars: Jahrom	28°49’ N	53°55’ E	1038	[65]	A range of decaying organic materials is exploited by this species [66]. Females oviposit on the edible paddy straw mushroom *Volvariella* (Plutaceae), as well as on rotting *Termitomyces* (Amanitaceae) [1]. It was also reported from bread fruits, *Artocarpus altilis* (Moraceae) [67], a colony of termite *Coptotermes niger* (Brues, 1925), the army ant *Eciton burchellii* and from the detritus from a tree hole [68].
	**Metopininae**							
2	*Arabiphora tenuifemorata* Disney, 2006	---	Fars: Jahrom	28°50’ N	53°56’ E	1020	[65]	nd
3	*Iranphora sharafkhaneensis* Namaki-Khameneh & Disney, 2021	(1–0)	East Azerbaijan: Sharafkhaneh	38°11.05’ N	45°29.52’ E	1313	[64]	nd
4	*Gymnophora arcuata (Meigen, 1830)	(1–0) (1–2)	East Azerbaijan: Bostan Abad, Qurigol region East Azerbaijan: Sharafkhaneh	37°54.736’ N 38°11.05’ N	46°41.617’ E 45°29.52’ E	1928 1313	[59] --	nd
5	*Megaselia albicaudata (Wood, 1910)	(2–0) (32–0)	East Azerbaijan: Arasbaran forest, Makidi East Azerbaijan: Sharafkhaneh Markazi	38°51.051’ N 38°11.05’ N --	46°54.892’ E 45°29.52’ E --	1406 1313 --	[62] “ [69]	Floral visitation [1]. Overwintering adults are found in caves [70,71].
6	*Megaselia altifrons* (Wood, 1909)	---	Markazi: Alborz Markazi: Bosri Markazi: Eybak abad Markazi: Far	34°00’09’’ N 33°48’32’’ N 34°16’55’’ N 34°25’32’’ N	49°20’34’’ E 48°58’24’’ E 49°36’51’’ E 49°12’36’’ E	1869 2000 1700 1900	[69] “ “ “	It was often found in forests and also on agricultural land [72–76]. The biology of the larvae is unknown; however, adults emerge from April to June [77].As a multivoltine species, the insect emergence period begins in spring and lasts until autumn [78].
7	*Megaselia aculeate (Schmitz, 1919)	(1–0) (1–0)	West Azerbaijan: Khoy, Evogli region West Azerbaijan: Mahabad	38°42.436’ N 36°34.16’ N	45°12.246’ E 45°41.21’E	968 1521	[61] ---	nd
8	*Megaselia albocingulata (Strobl, 1906)	(28–0) (730–0) (36–0) (340–0)	East Azerbaijan: Kandovan region East Azerbaijan: Sharafkhaneh West Azerbaijan: Khoy, Evogli region West Azerbaijan: Mahabad	37°46.10’ N 38°11.05’ N 38°42.436’ N 36°34.16’ N	46°16.001’ E 45°29.52’ E 45°12.246’ E 45°41.21’E	2500 1313 968 1521	[62] “ --- ---	Floral visitation [1].
9	*Megaselia ajabshirensis Namaki-Khameneh et al., 2019	(1–0) (1–0)	East Azerbaijan: Ajabshir region East Azerbaijan: Sharafkhaneh	37°31.978’ N 38°11.05’ N	46°07.716’ E 45°29.52’ E	1662 1313	[63] --	nd
10	*Megaselia angustiata Schmitz, 1936	(1–0) (240–0) (110–0) (20–0)	East Azerbaijan: Zonuz, Zonuzaq region East Azerbaijan: Sharafkhaneh West Azerbaijan: Mahabad West Azerbaijan: Khoy, Pere region	38°35.369’ N 38°11.05’ N 36°34.16’ N 38°36.722’ N	45°50.684’ E 45°29.52’ E 45°41.21’ E 44°53.336’ E	1758 1313 1521 1323	[62] -- -- --	Floral visitation [70].
11	*Megaselia ardabilensis Namaki-Khameneh et al., 2019	(10–0)	Ardabil: Meshgin Shahr, Ghirx-Bulax region	38°18.01’ N	47°23.02’ E	1803	[60]	nd
12	^●^*Megaselia angelicae* (Wood, 1910)	(1–0)	West Azerbaijan: Mahabad	36°34.16’ N	45°41.21’ E	1521	Current study	Floral visitation [1].
13	*Megaselia annulipes (Schmitz, 1921)	(4–0) (28–0)	West Azerbaijan: Khoy, Evogli region East Azerbaijan: Sharafkhaneh	38°42.436’ N 38°11.05’ N	45°12.246’ E 45°29.52’ E	968 1313	[60] --	nd
14	*Megaselia barzegarae Disney, 2012	(9–0) (6–0)	East Azerbaijan: Kandovan region West Azerbaijan: Miandoab region Kermanshah	37°46.10’ N 36°56.846’ N 34°19’21’’ N	46°16.001’ E 46°10.015’ E 47°05’21’’ E	2500 1306 1320	-- -- [79]	Reared from the fruiting bodies of agaric fungi [70].
15	*Megaselia brevicostalis (Wood, 1910)	(32–0) (8–0) (5–0) (820–0) (640–0)	West Azerbaijan: Khoy, Evogli region West Azerbaijan: Khoy, Pere region West Azerbaijan: Khoy, Pere region East Azerbaijan: Sharafkhaneh West Azerbaijan: Mahabad	38°42.436’ N 38°34.220’ N 38°36.722’ N 38°11.05’ N 36°34.16’ N	45°12.246’ E 44°50.896’ E 44°53.336’ E 45°29.52’ E 45°41.21’ E	968 1305 1323 1313 1521	[61] “ “ -- --	Larvae feed on dead insects/snails. Adults visit diverse flowers [70].
16	*Megaselia brevior (Schmitz, 1924)	(4–0) (36–0)	East Azerbaijan: Shabestar, Shanejan region East Azerbaijan: Sharafkhaneh	38°13.85’ N 38°11.05’ N	45°42.93’ E 45°29.52’ E	1664 1313	[62] “	Floral visitation [1].
17	*Megaselia bovista (Gimmerthal, 1848)	(6–0)	East Azerbaijan: Arasbaran forest, Makidi	38°51.051’ N	46°54.892’ E	1406	[62]	Oviposition onto the gills of mushroom sporophores [1,80].
18	^●^*Megaselia berndseni (Schmitz, 1919)	(11–0) (5–0)	East Azerbaijan: Sharafkhaneh West Azerbaijan: Mahabad	38°11.05’ N 36°34.16’ N	45°29.52’ E 45°41.21’ E	1313 1521	Current study	Compared to other phorids, larvae have been reported from sporophores of various fungi. Occasional floral visitation by adults [70].
19	*Megaselia coaetanea* Schmitz, 1929	---	Kermanshah: Koohsefid Kermanshah	35°02’38’’ N 34°19′27″ N	46°21’00’’ E 47°05′56″ E	1471 1323	[79] “	As a fungivorous fly, it was reared from the fruiting bodies of agaric mushroom, in Iran [70].
20	*Megaselia curvicapilla Schmitz, 1947	(8–0) (2400–0) (16–0)	East Azerbaijan: Zonuz, Mahar region East Azerbaijan: Sharafkhaneh East Azerbaijan: Arasbaran forest, Kaleybar region	38°39.339’ N 38°11.05’ N 38°51.548’ N	45°55.556’ E 45°29.52’ E 46°59.007’ E	2095 1313 1783	[62] “ --	nd
21	*Megaselia chicheckliensis Namaki-Khameneh et al., 2019	(4–0)	East Azerbaijan: Arasbaran forest, Chichekli region	38°39.899’ N	46°31.248’ E	2140	[63]	nd
22	*Megaselia caveonectergata Namaki-Khameneh & Disney, 2021	(2–0) (2–0)	East Azerbaijan: Arasbaran forest, Chichekli region East Azerbaijan: Sharafkhaneh	38°39.899’ N 38°11.05’ N	46°31.248’ E 45°29.52’ E	2140 1313	[64]	nd
23	^●^*Megaselia communiformis Schmitz, 1918	(5–0) (1–0)	East Azerbaijan: Sharafkhaneh East Azerbaijan: Sufiyan region	38°11.05’ N 38°17.01’ N	45°29.52’ E 46°10.02’ E	1313 1469	Current study	nd
24	*Megaselia distincta Namaki-Khameneh & Disney, 2021	(3–0) (1–0)	West Azerbaijan: Mahabad East Azerbaijan: Sharafkhaneh	36°34.16’ N 38°11.05’ N	45°41.21’ E 45°29.52’ E	1521 1313	[64]	nd
25	*Megaselia daemon* Bridarolli, 1951	---	Fars: Jahrom	28°53’ N	53°54’ E	1009	[65]	nd
26	*Megaselia exkaleybar Namaki-Khameneh et al., 2019	(3–0)	East Azerbaijan: Arasbaran forest, Kaleybar region	38°51.548’ N	46°59.007’ E	1783	[63]	nd
27	*Megaselia evogliensis Namaki-Khameneh et al., 2019	(2–0)	West Azerbaijan: Khoy, Evogli region	38°42.436’ N	45°12.246’ E	968	[63]	nd
28	*Megaselia fereagarici Namaki-Khameneh & Disney, 2021	(4–0)	West Azerbaijan: Mahabad	36°34.16’ N	45°41.21’ E	1521	[64]	nd
29	*Megaselia farshbafi* Namaki-Khameneh et al., 2019	(1–0)	West Azerbaijan: Khoy, Pere region	38°41.719’ N	44°54.041’ E	1405	[63]	nd
30	*Megaselia flavucrurus Namaki-Khameneh & Disney, 2021	(6–0)	West Azerbaijan: Mahabad	36°34.16’ N	45°41.21’ E	1521	[64]	nd
31	*Megaselia ghalateshahensis* Namaki-Khameneh et al., 2019	(1–0)	West Azerbaijan: Mahabad, Ghalate-Shah region	36°46.01’ N	46°22.37’ E	1605	[63]	nd
32	*Megaselia giraudii (Egger, 1862)	(18–0) (48–0)	West Azerbaijan: Khoy, Evogli region East Azerbaijan: Sharafkhaneh	38°42.436’ N 38°11.05’ N	45°12.246’ E 45°29.52’ E	968 1313	[61] --	The common saprophage in bird nests [81]. Breeding in fungus sporophores [1]. Larvae are common scavengers on decaying organic matters, but especially on dead insects. Occasional floral visitation by adults [70].
33	*Megaselia haddadi Namaki-Khameneh et al., 2019	(2–0)	West Azerbaijan: Khoy, Evogli region	38°42.436’ N	45°12.246’ E	968	[63]	nd
34	*Megaselia halterata (Wood, 1910)	(2–0) (22–0)	West Azerbaijan: Khoy, Evogli region East Azerbaijan: Sharafkhaneh Alborz: Karaj Kermanshah	38°42.436’ N 38°11.05’ N -- --	45°12.246’ E 45°29.52’ E -- --	968 1313-- --	[61] -- [82] [83]	Larvae as important pest of the button mushrooms in Iran and other areas [84,85]. A phorid with known bionomics in Iran [50].
35	*Megaselia hejazii Namaki-Khameneh et al., 2019	(2–0) (6–0)	West Azerbaijan: Khoy, Pere region East Azerbaijan: Sharafkhaneh	38°36.722’ N 38°11.05’ N	44°53.336’ E 45°29.52’ E	1323 1313	[63] --	nd
36	*Megaselia hirticaudata (Wood, 1910)	(1–0) (2–0)	West Azerbaijan: Khoy, Evogli region East Azerbaijan: Bostan abad, Qurigol region	38°42.436’ N 37°54.736’ N	45°12.246’ E 46°41.617’ E	968 1928	[61] --	nd
37	*Megaselia hendersoni* Disney, 1979	(1–0)	West Azerbaijan: Khoy, Pere region	38°41.719’ N	44°54.041’ E	1405	[61]	nd
38	^●^*Megaselia hirtiventris* (Wood, 1909)	(1–0)	East Azerbaijan: Sharafkhaneh	38°11.05’ N	45°29.52’ E	1313	Current study	Fungus-feeding larvae [1].
39	*Megaselia khoyensis Namaki-Khameneh et al., 2019	(24–0)	West Azerbaijan: Khoy, Evogli region	38°42.436’ N	45°12.246’ E	968	[63]	nd
40	*Megaselia khaghaniniai* Namaki-Khameneh & Disney 2019	(1–0)	West Azerbaijan: Khoy, Pere region	38°41.719’ N	44°54.041’ E	1405	[63]	nd
41	*Megaselia kermanshahensis* Disney, 2012	---	Kermanshah Kermanshah: Sahne Park Kermanshah: Sarab Qanbar Kermanshah: Haft Ashiyan Village	34°19’27’’ N 34°29′07″ N 34°17′12″ N 34°58′04″ N	47°05’56’’ E 47°41′39″ E 47°03′17″ E 47°27′54″ E	1323 1401 1461 1805	[79] “ “ “	As a fungivorous fly, it was reared from the fruiting bodies of agaric mushroom, in Iran [79].
42	*Megaselia kaleybarensis Namaki-Khameneh et al., 2019	(47–0) (30–0)	East Azerbaijan: Arasbaran forest, Kaleybar region West Azerbaijan: Khoy, Evogli region	38°51.548’ N 38°42.436’ N	46°59.007’ E 45°12.246’ E	1783 968	[63] --	nd
43	*Megaselia longiseta (Wood, 1909)	(1–0) (3–0)	East Azerbaijan: Arasbaran forest, Kaleybar region East Azerbaijan: Sharafkhaneh	38°51.548’ N 38°11.05’ N	46°59.007’ E 45°29.52’ E	1783 1313	[62] “	nd
44	*Megaselia ledzona Namaki-Khameneh et al., 2019	(49–0)	West Azerbaijan: Khoy, Evogli region	38°42.436’ N	45°12.246’ E	968	[63]	nd
45	^●^*Megaselia longipalpis (Wood, 1910)	(4–0)	West Azerbaijan: Mahabad	36°34.16’ N	45°41.21’ E	1521	Current study	nd
46	^●^*Megaselia largifrontalis* Schmitz, 1939	(1–0)	East Azerbaijan: Arasbaran forest, Chichekli region	38°39.899’ N	46°31.248’ E	2140	Current study	nd
47	*Megaselia mahabadensis* Namaki-Khameneh et al., 2019	(1–0)	West Azerbaijan: Mahabad, Ghalate-Shah region	36°46.01’ N	46°22.37’ E	1605	[63]	nd
48	*Megaselia minuta (Aldrich, 1892)	(2–0) (8–0)	West Azerbaijan: Khoy, Pere region West Azerbaijan: Mahabad	38°34.220’ N 36°34.16’ N	44°50.896’ E 45°41.21’ E	1305 1521 m	[61] --	Coexist in the nests of wrens, *Troglodytes troglodytes* [1].
49	*Megaselia meconicera* (Speiser, 1925)	(1–0)	West Azerbaijan: Khoy, Evogli region	38°42.436’ N	45°12.246’ E	968	[61]	Larvae found in mammalian dung and adults in large numbers in houses in the cold months [1].
50	*Megaselia minor* (Zetterstedt, 1848)		Markazi: Bazeneh	34°02’32’’ N	49°23’26’’ E	2000	[69]	In Europe, the species was often collected in forests [e. g. 72,86]. As a multivoltine species, the insect emergence period begins in spring and lasts until autumn [78]. Occasional floral visitation by adults [70].
51	*Megaselia miandoabensis Namaki-Khameneh et al., 2019	(5–2)	West Azerbaijan: Miandoab region	36°56.846’ N	46°10.015’ E	1306	[63]	nd
52	*Megaselia namakiae Khaghaninia & Disney, 2019	(1–0) (1–0)	West Azerbaijan: Khoy, Evogli region East Azerbaijan: Kaleybar, Khodaafarin region	38°42.436’ N 39°09.93’ N	45°12.246’ E 47°01.07’ E	968 237	[63] --	nd
53	*Megaselia oxybelorum Schmitz, 1928	(2–0) (4–0) (31–0)	East Azerbaijan: Shabestar, Shanejan region Ardabil: Meshgin Shahr, Ghirx-Bulax region East Azerbaijan: Sharafkhaneh	38°13.85’ N 38°18.01’ N 38°11.05’ N	45°42.93’ E 47°23.02’ E 45°29.52’ E	1664 1803 1313	[62] -- --	Parasitoids/reared of/from the fly *Fannia scalaris* (Fanniidae) and the wasps of the *Cerceris arenaria*, *Oxybelus uniglumis* (Crabronidae) *Philanthus triangulum* (Philanthidae) [4,87–89].
54	*Megaselia plurispinulosa (Zetterstedt, 1860)	(6–6) (5–0)	West Azerbaijan: Khoy, Evogli region East Azerbaijan: Sharafkhaneh	38°42.436’ N 38°11.05’ N	45°12.246’ E 45°29.52’ E	968 1313	[61] --	Larvae are fungivorous on *Boletus edulis*, *Boletus pinophilus* (Boletaceae), and *Pleurotus cornucopiae* (Lentinaceae), as well as adults flower visitors of *Anthriscus sylvestris* (Apiaceae) [70].
55	*Megaselia pereensis* Namaki-Khameneh et al., 2019	(1–0)	West Azerbaijan: Khoy, Pere region	38°41.719’ N	44°54.041’ E	1405	[63]	nd
56	*Megaselia product (Schmitz, 1921)	(1–0) (1–0)	Ardabil: Meshgin Shahr, Geyneje region West Azerbaijan: Mahabad	38°20.37’ N 36°34.16’ N	47°48.66’ E 45°41.21’ E	2991 1521	[60] --	nd
57	*Megaselia pleuralis (Wood, 1909)	(16–0)	West Azerbaijan: Khoy, Evogli region	38°42.436’ N	45°12.246’ E	968	[61]	Larvae found in pigeon dung, and adults are visitors a a wide range of flower species [70].
58	^●^*Megaselia propinqua (Wood, 1909)	(6–0)	East Azerbaijan: Sharafkhaneh	38°11.05’ N	45°29.52’ E	1313	Current study	Floral visitation [1].
59	^●^*Megaselia pusilla (Meigen, 1830)	(1100–0) (900–0) (61–0)	East Azerbaijan: Sharafkhaneh West Azerbaijan: Mahabad West Azerbaijan: Khoy, Pere region	38°11.05’ N 36°34.16’ N 38°41.719’ N	45°29.52’ E 45°41.21’ E 44°54.041’ E	1313 1521 1405	Current study	nd
60	*Megaselia posticata (Strobl, 1898)	(2–0) (3–0)	East Azerbaijan: Bostan abad, Qurigol region East Azerbaijan: Sharafkhaneh	37°54.736’ N 38°11.05’ N	46°41.617’ E 45°29.52’ E	1928 1313	[62] “	Floral visitation [1].
61	^●^*Megaselia perdistans (Schmitz, 1924)	(2–0) (1–0)	East Azerbaijan: Sharafkhaneh West Azerbaijan: Mahabad	38°11.05’ N 36°34.16’ N	45°29.52’ E 45°41.21’ E	1313 1521	Current study	nd
62	*Megaselia polysetosis Namaki-Khameneh & Disney, 2021	(3–0) (46–0)	East Azerbaijan: Sharafkhaneh West Azerbaijan: Mahabad	38°11.05’ N 36°34.16’ N	45°29.52’ E 45°41.21’ E	1313 1521	[64]	nd
63	*Megaselia paluventer Namaki-Khameneh & Disney, 2021	(41–0)	East Azerbaijan: Sharafkhaneh	38°11.05’ N	45°29.52’ E	1313	[64]	nd
64	^●^*Megaselia pallidizona (Lundbeck 1920)	(48–0)	East Azerbaijan: Sharafkhaneh	38°11.05’ N	45°29.52’ E	1313	Current study	nd
65	*Megaselia qurigolensis Namaki-Khameneh et al., 2019	(2–0) (1–0)	East Azerbaijan: Bostan abad, Qurigol region West Azerbaijan: Mahabad	37°54.736’ N 36°34.16’ N	46°41.617’ E 45°41.21’ E	1928 1521	[63] --	nd
66	^●^*Megaselia ruficornis (Meigen, 1830)	(2–0)	East Azerbaijan: Sharafkhaneh	38°11.05’ N	45°29.52’ E	1313	Current study	Larvae are scavengers on decaying organic matters (dead molluscs/insects, dung and vertebrate meat baits) and females fungivorous [70].
67	*Megaselia rufipes (Meigen, 1804)	(15–0) (20–0) (15–0)	East Azerbaijan: Ajabshir region East Azerbaijan: Sharafkhaneh West Azerbaijan: Mahabad Fars: Jahrom	37°31.978’ N 38°11.05’ N 36°34.16’ N 28°50’ N	46°07.716’ E 45°29.52’ E 45°41.21’ E 53°55’ E	1662 1313 1521 1040	[62] “ -- [65]	Larvae are scavengers on decaying broad spectrum of organic matters (rotting plants, dung, decaying fungi, dead invertebrates, and vertebrate carrion, including human corpses). They occasionally exploit human foods, such as cheese and rice-based pre-cooked meals. Adults visit a variety of flowers and fungus spores have been found in the crops of females [70].
68	*Megaselia styloprocta (Schmitz, 1921)	(2–0)	East Azerbaijan: Zonuz, Zonuzaq region	38°35.369’ N	45°50.684’ E	1758	[62]	nd
69	*Megaselia shabestarensis Namaki-Khameneh et al., 2019	(38–0)	East Azerbaijan: Shabestar, Shanejan region	38°13.85’ N	45°42.93’ E	1664	[63]	nd
70	*Megaselia subnudipennis (Schmitz, 1919)	(2–0) (12–0)	West Azerbaijan: Miandoab region East Azerbaijan: Sharafkhaneh	36°56.846’ N 38°11.05’ N	46°10.015’ E 45°29.52’ E	1306 1313	[61] --	Floral visitation [1].
71	*Megaselia scalaris* Loew, 1866		Fars: Jahrom Mazandaran: Amol, Ghaemshahr, Behshahr Tehran: Shahr-e-rey Golestan: Gorgan Alborz: Karaj Zanjan	28°50’ N -- -- -- -- -- --	53°54’ E -- -- -- -- -- --	1018 -- -- -- -- -- --	[49] [90] “ “ [51] [24]	Larvae are polyphagous. Their favoring substrates include decaying coconuts [91], ripe bananas [92] and dead rabbits [1]. However, they were reported from human corpses [93] and near dirty floor-drains and mausoleums [94]. This species is recognized as an important pest of the button mushrooms in Karaj mushroom houses [49]. Also, it invades insect cultures [90] and parasites asilid species and honey bee colonies [51] in Iran. *M*. *scalaris* is reported to be a cause of myiasis on humans and animals [24,95,96].
72	*Megaselia subfuscipes* Schmitz, 1935		Markazi: Cheshme sar Fars: Jahrom	34°07’57’’ N 28°53’ N	49°10’48’’ E 53°54’ E	2098 1009	[69] [65]	They are most often reported from agricultural lands in Germany [73,86,97]. Larvae considered to be zoosaprophagous [86].
73	*Megaselia stichata (Lundbeck, 1920)	(1–0) (14–0) (10–0)	East Azerbaijan: Kandovan region East Azerbaijan: Sharafkhaneh West Azerbaijan: Mahabad	37°46.10’ N 38°11.05’ N 36°34.16’ N	46°16.001’ E 45°29.52’ E 45°41.21’ E	2500 1313 1521	[62] “ --	nd
74	^●^*Megaselia sandhui Disney, 1981	(7–0) (15–0)	East Azerbaijan: Arasbaran forest, Chichekli region East Azerbaijan: Sufiyan region	38°39.899’ N 38°17.01’ N	46°31.248’ E 46°10.02’ E	2140 1469	Current study	nd
75	^●^*Megaselia stigmatica* (Schmitz, 1920)	(1–0)	West Azerbaijan: Mahabad	36°34.16’ N	45°41.21’ E	1521	Current study	nd
76	^●^*Megaselia subpleuralis* (Wood, 1909)	(1–0)	West Azerbaijan: Mahabad	36°34.16’ N	45°41.21’ E	1521	Current study	nd
77	^●^*Megaselia spinicincta (Wood, 1910)	(3–0) (12–0)	East Azerbaijan: Sharafkhaneh West Azerbaijan: Mahabad	38°11.05’ N 36°34.16’ N	45°29.52’ E 45°41.21’ E	1313 1521	Current study	Fungus-feeding larvae [1].
78	*Megaselia tama (Schmitz, 1926)	(1–0) (1–0)	East Azerbaijan: Arasbaran forest, Makidi East Azerbaijan: Sharafkhaneh	38°51.051’ N 38°11.05’ N	46°54.892’ E 45°29.52’ E	1406 1313	[62] “	nd
79	^●^*Megaselia tarsalis (Wood, 1910)	(3–0) (11–0)	East Azerbaijan: Sharafkhaneh East Azerbaijan: Shabestar, Shanejan region	38°11.05’ N 38°13.85’ N	45°29.52’ E 45°42.93’ E	1313 1664	Current study	nd
80	^●^*Megaselia verna Schmitz, 1932	(6–0) (28–0)	East Azerbaijan: Sharafkhaneh West Azerbaijan: Mahabad	38°11.05’ N 36°34.16’ N	45°29.52’ E 45°41.21’ E	1313 1521	Current study	nd
81	*Megaselia verralli (Wood, 1910)	(52–0) (37–0) (1960–0) (1830–0)	West Azerbaijan: Khoy, Evogli region West Azerbaijan: Khoy, Pere region East Azerbaijan: Sharafkhaneh West Azerbaijan: Mahabad	38°42.436’ N 38°41.719’ N 38°11.05’ N 36°34.16’ N	45°12.246’ E 44°54.041’ E 45°29.52’ E 45°41.21’ E	968 1405 1313 1521	[61] “ -- --	nd
82	*Megaselia xanthozona (Strobl, 1892)	(9–0) (760–0) (14–0) (3–0) (570–0) (4–0)	East Azerbaijan: Shabestar, Shanejan region East Azerbaijan: Sharafkhaneh West Azerbaijan: Khoy, Evogli region West Azerbaijan: Khoy, Pere region West Azerbaijan: Mahabad East Azerbaijan: Arasbaran forest, Kaleybar region Markazi: Hafteh Markazi: Shanagh Fars: Jahrom	38°13.85’ N 38°11.05’ N 38°42.436’ N 38°34.220’ N 36°34.16’ N 38°51.548’ N 33°59’49’’ N 33°58’06’’ N 28°50’ N	45°42.93’ E 45°29.52’ E 45°12.246’ E 44°50.896’ E 45°41.21’ E 46°59.007’ E 49°23’58’’ E 50°24’10’’ E 53°53’ E	1664 1313 968 1305 1521 1783 1588 2143 1028	[62] “ [61] “ -- [69] “ [65]	In Europe, it is often collected from forests [72,76], as well as in the Alpine zone of the Alps [98]. Adults visit flowers of *Gypsophila hispanica* (Caryophyllaceae) [99].
83	*Megaselia yaseri* Namaki-Khameneh et al., 2019	(1–0)	West Azerbaijan: Khoy, Pere region	38°36.722’ N	44°53.336’ E	1323	[63]	nd
84	*Megaselia zarghanii Namaki-Khameneh et al., 2019	(3–0)	West Azerbaijan: Mahabad, Ghalate-Shah region	36°46.01’ N	46°22.37’ E	1605	[63]	nd
85	*Megaselia zonuzensis Namaki-Khameneh et al., 2019	(23–0) (45–0) (20–0) (15–0)	East Azerbaijan: Zonuz, Zonuzaq region East Azerbaijan: Sharafkhaneh West Azerbaijan: Khoy, Evogli region West Azerbaijan: Khoy, Pere region	38°35.369’ N 38°11.05’ N 38°42.436’ N 38°34.220’ N	45°50.684’ E 45°29.52’ E 45°12.246’ E 44°50.896’ E	1758 1313 968 1305	[63] -- -- --	nd
86	*Metopina heselhausi Schmitz 1914	(15–0)	East Azerbaijan: Sharafkhaneh Fars: Jahrom Fars: Jahrom	38°11.05’ N 28°53’ N 28°54’ N	45°29.52’ E 53°54’ E 53°53’ E	1313 1009 996	-- [65] “	Adults visit the flowers of *Taraxacum officinale* (Asteraceae), *Reseda lutea* (Resedaceae), and *Potentilla anserina* (Rosaceae), as well as meat baits [70].
87	*Metopina oligoneura* (Mik, 1867)	(1–0)	West Azerbaijan: Khoy, Evogli region	38°42.436’ N	45°12.246’ E	968	[59]	Floral visitation. Saprophage species exploiting from the decaying or locally damaged leaves/roots of beet. Reared from meat bait buried 15–20 cm in arable soil [1].
88	^●^*Metopina perpusilla (Six, 1878)	(12–0)	East Azerbaijan: Sharafkhaneh	38°11.05’ N	45°29.52’ E	1313	Current study	nd
89	*Phalcrotophora flavidus* Namaki-Khameneh & Disney, 2021	(1–0)	East Azerbaijan: Sharafkhaneh	38°11.05’ N	45°29.52’ E	1313	[64]	nd
90	*Phalacrotophora fasciata* (Fallen, 1823)		Mashhad	--	--	--	[100]	It was reported as pupal parasitoid on seven-spot ladybird, in Mashhad, in 2012 [100].
	**Phorinae**							
91	*Conicera tibialis Schmitz, 1925	(6–0) (10–0)	West Azerbaijan: Khoy, Evogli region East Azerbaijan: Sharafkhaneh	38°42.436’ N 38°11.05’ N	45°12.246’ E 45°29.52’ E	968 1313	[59] --	The larvae feed on buried human corpses. They are also scavengers in wasp and bird nests. Adults visit a range of flowers in Apiaceae [70].
92	*Diplonevra funebris (Meigen, 1830)	(9–3) (6–0) (12–4)	Ardabil: Meshgin Shahr, Ghirx-Bulax region West Azerbaijan: Khoy, Evogli region East Azerbaijan: Sharafkhaneh Markazi: Tooreh	38°18.01’ N 38°42.436’ N 38°11.05’ N 34°06’41’’ N	47°23.02’ E 45°12.246’ E 45°29.52’ E 49°19’19’’ E	1803 968 1313 1939	[60] -- -- [69]	The larvae are zoosaprophagous, chiefly on animal carrion (pork, snail, duck and sheep) [1,86]. The larvae feed on dead invertebrates and have frequently been recorded as scavengers in wasp nests. Adults visit a range of flowers in Apiaceae and Asteraceae [70].
93	*Dohrniphora cornuta (Bigot, 1857)	(1–0) (1–0)	East Azerbaijan: Kandovan region East Azerbaijan: Kaleybar, Khodaafarin region	37°46.10’ N 39°09.93’ N	46°16.001’ E 47°01.07’ E	2500 237	[59] --	Larvae are scavengers on decaying organic matters (rotten potatoes, onions, beans, rice bran, chick peas, dead insects, dead snails, sour milk, human faeces, general garbage, dead mice and other small mammals, moribund eggs of turtles, and the sewage film of micro-organisms in trickling filter sewage beds). Adults visit flowers of Aristolochia as a major pollinator in Thailand[70,101].
94	*Phora holosericea Schmitz, 1920	(16–6) (5–2) (4–2) (18–9) (12–4)	Ardabil: Meshgin Shahr, Geyneje region East Azerbaijan: Sufiyan region West Azerbaijan: Mahabad, Ghalate-Shah region East Azerbaijan: Sharafkhaneh West Azerbaijan: Mahabad Markazi: Aman abad Markazi: Akbar abad Markazi: Gvar Markazi: Mahdi abad Markazi: Vismeh Markazi: Zarrin khoshe	38°20.37’ N 38°17.01’ N 36°46.01’ N 38°11.05’ N 36°34.16’ N 34°03’27’’ N 34°48’32’’ N 34°01’40’’ N 34°06’07’’ N 34°12’28’’ N 35°30’11’’ N	47°48.66’ E 46°10.02’ E 46°22.37’ E 45°29.52’ E 45°41.21’ E 49°46’49’’ E 50°16’26’’ E 49°17’47’’ E 49°19’40’’ E 50°02’53’’ E 50°24’00’’ E	2991 1469 1605 1313 1521 1800 1850 2000 1900 1700 1697	[60] -- -- -- -- [69] “ “ “ “ “	Larvae feed on Aphids as predators [1].
95	*Phora iranensis Namaki-Khameneh & Disney, 2021	(4–0)	West Azerbaijan: Mahabad	36°34.16’ N	45°41.21’ E	1521	[64]	nd
96	#*Mahabadphora aesthesphora Namaki-Khameneh & Disney, 2021	(2–0)	West Azerbaijan: Mahabad	36°34.16’ N	45°41.21’ E	1521		nd
97	*Triphleba intermedia (Malloch, 1908)	(5–0)	East Azerbaijan: Sufiyan region	38°17.01’ N	46°10.02’ E	1469	[59]	Floral visitation [1].
		(13903–40)						

Symbols: ^●^ New record for Iran

* Species with molecular data

^#^ New genus.

Note: There is no way or identification key to discriminate the female specimens of most species; as a result, the majority of females (n = 8057) were not included in the table.

nd: Not defined.

**Description of a new genus and species.** *Taxonomy*

### Subfamily Phorinae

***Mahabadphora*** Namaki-Khameneh & Disney n. gen.

LSID: urn:lsid:zoobank.org:act:BB5DCD6D-EFA2-4E34-828A-C34A1438E093

#### Diagnosis (male)

In the key to world genera of the Phoridae [1], it runs to couplet 73 lead 1 *Chaetopleurophora* Schmitz. It is immediately distinguished by its anal tube being very much longer than the epandrium as opposed to being very much shorter. Its globose postpedicels its costal index being less than 0.4 further distinguishes the new genus. It fails to run to *Rhynchomicropteron* Annandale, 1912 in the key to the males. This genus is primarily known from the females, with their greatly reduced wings. However, Lengyel [102] has provided a well illustrated key to a male. His Fig 6 of the very distinctive frons, whose breadth is about twice its length, immediately distinguished from *Mahabadphora*.

#### Etymology

Named after the city Mahabad (locality of the holotype).

***Mahabadphora aesthesphora*** Namaki-Khameneh & Disney n. sp.

LSID: urn:lsid:zoobank.org:act:4FD302BB-CBAF-45E2-B2D2-D53F6DF1DCA0

#### Specification (male)

**Fig 1:**
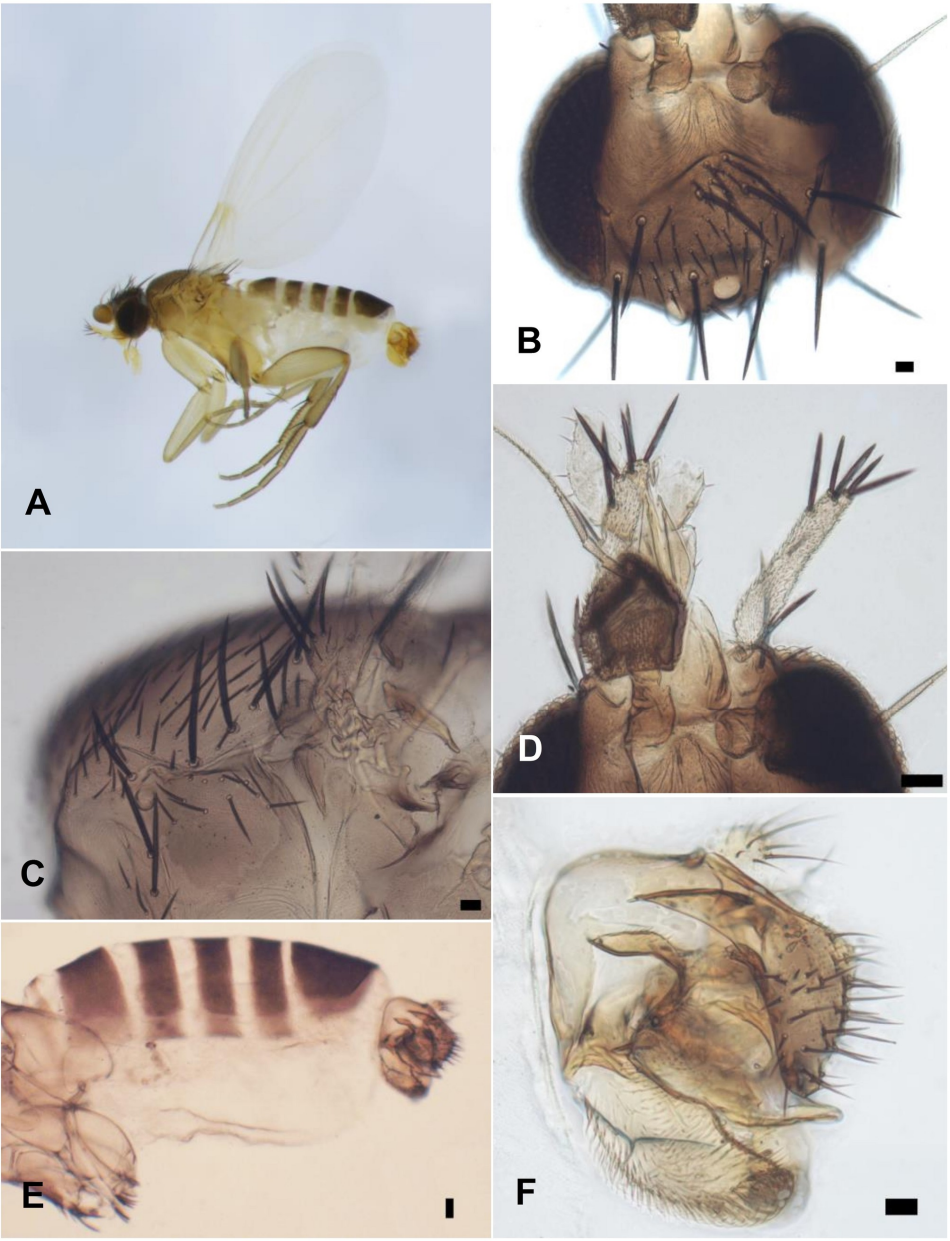
*Mahabadphora aesthesphora* n. gen, n. sp., male. A, whole fly; B, frons; C, side of thorax; D, antennae, palps, and proboscis; E, abdomen; F, left face of hypopygium. The right black rectangle of each image represents a scale of 20 μm.

**Fig 2:**
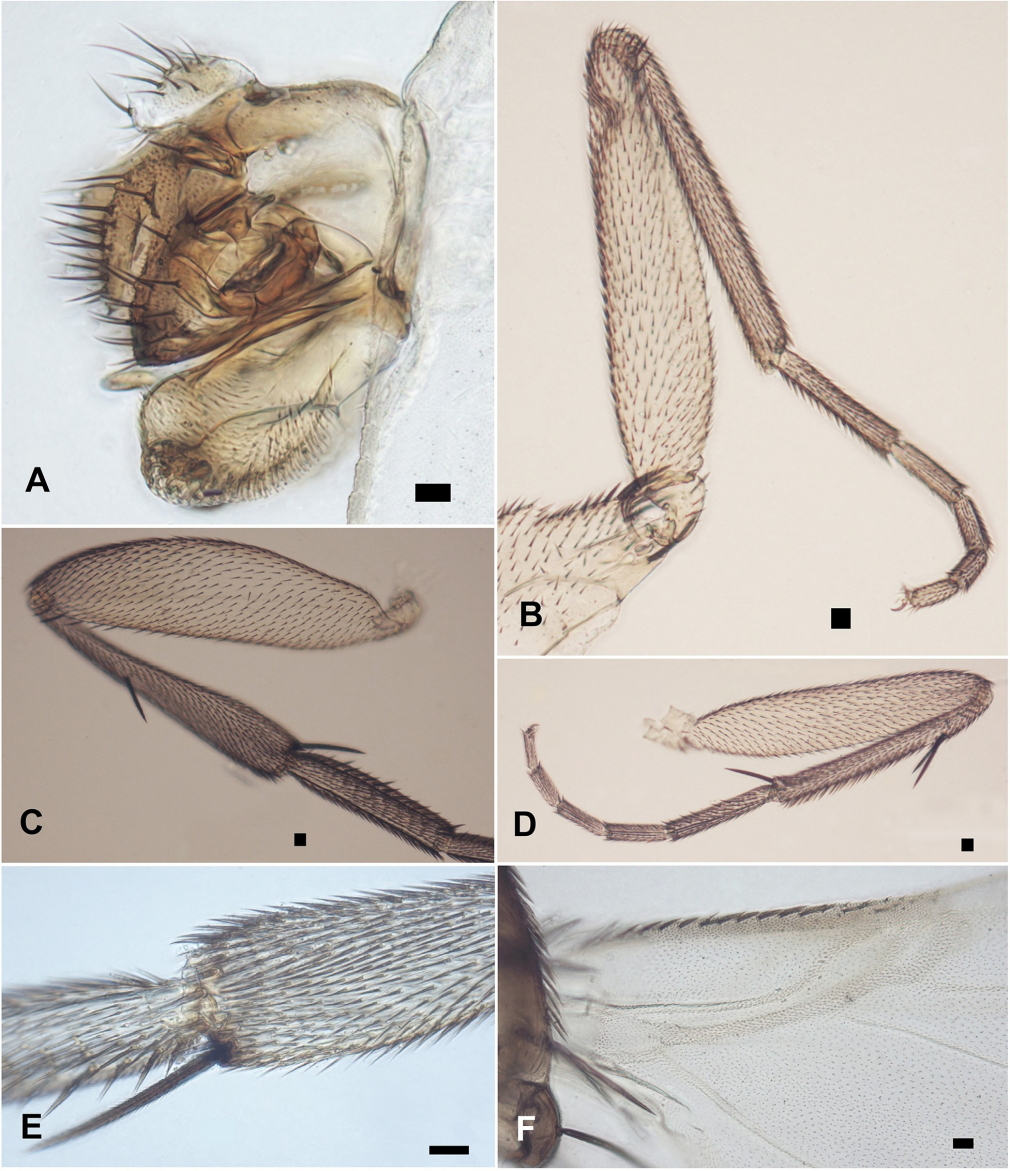
*Mahabadphora aesthesphora* n. gen, n. sp., male. A, right face of hypopygium; B, front leg; C, hind femur, tibia and basitarsus; D, middle leg; E, postereior face of tip of hind tibia; F, basal third of wing. The right black rectangle of each image represents a scale of 20 μm.

Fig 1A, whole fly. Frons as Fig 1B, without a median furrow, with a pair of short supra-antennal bristles and 4–4 long bristles. Side of thorax as Fig 1C, the mesopleuron with hairs. Antennae, palps, and proboscis as Fig 1D. Scutellum with a pair of long bristles and a pair of short hairs. Abdomen as Fig 1E, the hairs of the tergites being very small and the venter lacking hairs. The left face of hypopygium as Fig 1F and the right face of hypopygium as Fig 2A, being notable for the strong bristles on the epandrium, the small left lobe of the hypandrium and huge right lobe. Front leg as Fig 2B. Middle leg as Fig 2D. Hind femur, tibia, and basitarsus as Fig 2C, the tibia without a dorsal hair palisade and the postereior face of tip of hind tibia as Fig 2E. Very pale wing, basal third of wing as Fig 2F. Wing length 1.44 mm. Costal index 0.31. Costal ratios about 5: 1.3: 1. Costal cilia 0.02 mm long. The single axillary bristle 0.05 mm long. Haltere very pale.

#### Material examined

Holotype male, Iran, West Azerbaijan, Mahabad City, 36°34.16’N, 45°41.21’E, 1521m, 23.VII.2018. Samad Khaghaninia (71, UCMZ, 13–104).

#### Etymology

Named after it being strange (Greek aesthes).

#### Ecological data

**Fig 3:**
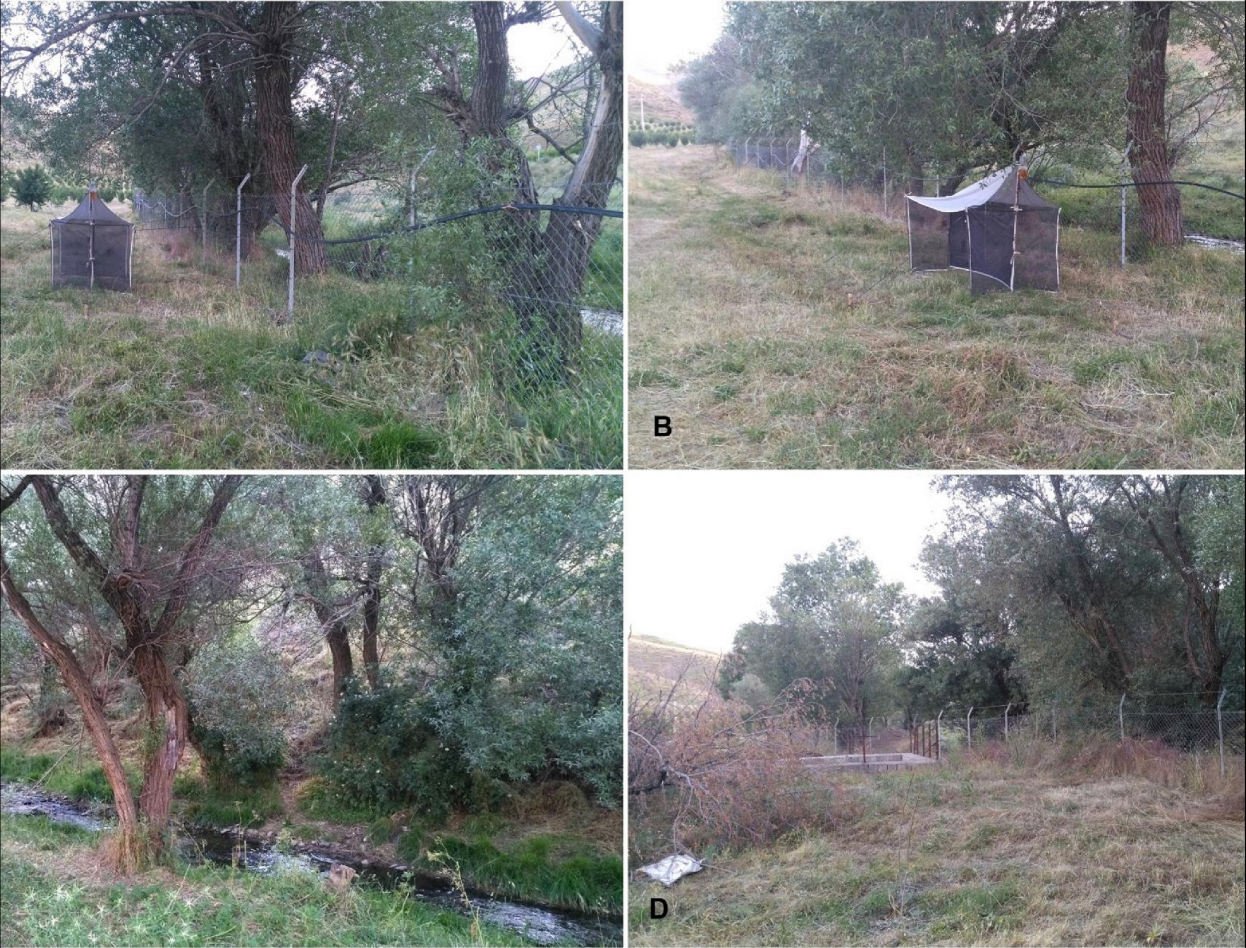
Natural habitats for *Mahabadphora aesthesphora* n. gen, n. sp. in the Mahabad City.

The climate at the type locality is temperate with very cold winters and hot summers. The sampling site is located within the valley, which has a seasonal river running until the end of July. Herbaceous and woody vegetation plants in the area comprises of *Glycyrrhiza glabra* L. (Fabaceae), *Achillea millefolium* L. (Asteraceae), *Peganum harmala* L. (Zygophyllaceae), *Convolvulus arvensis* L. (Convolvulaceae), and *Salix alba* L.) Salicaceae (etc. In general, the region has previously been quite untouched and pristine, but recently, it has been modified or influenced by human activities (Fig 3).

### Molecular surveys

#### Sequence analysis

Two *COI* and *28S* genes for all studied specimens and three *COI*, *28S* and *AK* genes for the new genus were successfully amplified and sequenced (S1 Table). In total, 720, 512–549, and 543 base pairs were sequenced for the *COI*, *28S*, and *AK* genes of the studied specimens, respectively. The corresponding sequences were deposited in the GenBank under accession numbers MN597118-MN597188 (*COI*), MN833420-MN833490 (*28S*), and MN723164 (*AK*). The multiple sequence alignments showed relatively a large number of substitutions and a few indels among the *COI*/*AK* and *28S* gene sequences, correspondingly. The analysis of the *COI* sequences revealed 349 (49%) polymorphic sites, where 310 (43%) was the parsimony-informative site. There were also 104 (19%) polymorphic sites with 60 (11%) parsimony-informative sites in the *28S* gene sequences, in comparison. Among 543-bp *AK* gene analyzed, 166 (30.57%) were polymorphic sites, and 101 (18.60%) were parsimony-informative.

#### Taxa and genetic diversities

Seventy-one morphologically defined species belonged to nine genera, comprising of *Conicera* Meigen, 1830 (n = 1), *Diplonevra* Lioy, 1864 (n = 1), *Dohrniphora* Dahl, 1898 (n = 1), *Gymnophora* Macquart, 1835 (n = 1), *Mahabadphora* (n = 1), *Megaselia* Rondani, 1856 (n = 61), *Metopina* Macquart, 1835 (n = 2), *Phora* Latreille, 1796 (n = 2), and *Triphleba* Rondani, 1856 (n = 1), as shown in Table 2. All 71 species studied were readily differentiated from each other by the *COI* (but not *28S*) sequences (data are not shown). The molecular analysis of *COI* sequences did not display any dissimilarity between the members of the two pairs of *Megaselia* species, i.e. *M*. *hirticaudata* / *M*. *shabestarensis* and *M*. *khoyensis* / *M*. *ardabilensis*. Based on the K2P substitution model, the average interspecific pairwise distances among *COI*, *28S*, and *AK* sequences were determined as 16.67%, 1.39%, and 10.76%, respectively (Tables 3, S3 and S4). The mean intergeneric genetic divergence of *COI*, *28S*, and *AK* sequences was calculated correspondingly as 17.30%, 1.29% and 10.76%, respectively (Tables 3–6). For all the target genes, the genetic distances of *Mahabadphora* from all studied genera were greater than the mean intergeneric divergence estimated (shown by the shaded column/row in Tables 3–5). The average intrageneric genetic distances of the analyzed *COI* sequences varied from 8.06% (*Stichillus* Enderlein 1924) to 15.63% (*Megaselia*), which were negligible for the specimens studied based on *28S* gene, which ranged from 0.05% (*Anevrina* Lioy, 1864) to 0.77% (*Megaselia*). Besides, for the *AK* gene, the distance was in the range from 4.16% (*Myriophora* Brown, 1992) to 5.41% (*Apocephalus* Coquillett, 1901).

**Table 3:** K2P genetic distances ± standard deviations (SD) for studied scuttle flies genera based on 720 bps of *COI* sequences.

Group	Mean intergeneric genetic divergence ± SD (min–max)																				mean intrageneric genetic divergence ±SD (min–max)
	1	2	3	4	5	6	7	8	9	10	11	12	13	14	15	16	17	18	19	20	
1. Conicera (n = 1)																					nc
2. Gymnophora (n = 2)	18.21±1.61 (16.40–20.01)																				14.54
3. Triphleba (n = 1)	15.88±1.53 (15.88–15.88)	19.04±1.64 (17.85–20.23)																			nc
4. Dohrniphora (n = 13)	16.79±1.35 (15.52–18.41)	18.40±1.36 (15.88–23.30)	16.09±1.27 (14.32–18.04)																		11.70±0.82 (0.14–16.56)
5. Phora (n = 2)	21.79±1.87 (21.58–21..99)	23.36±1.74 (22.13–24.66)	23.22±1.91 (23.20–23.23)	23.09±1.71 (21.78–24.95)																	15.96
6. Diplonevra (n = 6)	16.42±1.33 (14.83–19.28)	17.43±1.32 (15.87–19.29)	16.27±1.31 (15.01–17.30)	14.30±1.00 (10.80–17.29)	22.87±1.72 (21.35–23.54)																11.64±0.88 (4.02–14.01)
7. Metopina (n = 2)	19.84±1.68 (19.65–20.03)	18.69±1.47 (17.70–20.06)	20.61±1.68 (20.43–20.79)	18.12±1.35 (16.07–22.73)	24.97±1.88 (23.26–27.09)	18.34±1.42 (16.94–20.05)															12.92
8. Mahabadphora (n = 1)	18.37±1.75 (18.37–18.37)	20.58±1.63 (20.01–21.14)	20.20±1.81 (20.20–20.20)	18.89±1.40 (17.11–20.38)	24.43±1.93 (22.66–26.19)	18.54±1.44 (17.31–20.40)	22.85±1.82 (21.90–23.80)														nc
9. Megaselia (n = 65)	18.60±1.34 (15.18–21.70)	15.86±1.08 (12.47–21.73)	18.30±1.27 (14.69–21.16)	17.37±1.09 (11.95–23.24)	22.86±1.57 (19.85–27.89)	16.96±1.08 (12.12–22.85)	18.18±1.25 (15.39–23.29)	19.67±1.33 (15.87–22.66)													15.63±0.92 (0–22.66)
10. Apodicrania (n = 1)	18.55±1.75 (18.55–18.55)	18.40±1.55 (16.40–20.40)	18.93±171 (18.93–18.93)	17.97±1.38 (15.88–20.96)	22.79±1.88 (20.95–24.63)	18.14±1.42 (16.55–21.32)	18.11±1.60 (17.47–18.74)	19.84±1.74 (19.84–19.84)	16.26±1.15 (13.63–19.85)												nc
11. Melaloncha (n = 1)	20.43±1.81 (20.43–20.43)	19.69±1.52 (18.76–20.61)	23.10±1.95 (23.10–23.10)	21.12±1.59 (19.49–22.13)	22.31±1.81 (21.92–22.69)	21.29±1.61 (20.23–22.68)	22.92±1.78 (22.12–23.72)	20.95±1.87 (20.95–20.95)	20.57±1.41 (17.00–24.42)	21.36±1.76 (21.36–21.36)											nc
12. Phalacrotophora (n = 1)	21.73±2.02 (21.73–21.73)	21.52±1.81 (21.25–21.80)	22.18±2.06 (22.18–22.18)	20.46±1.61 (18.57–22.11)	25.44±2.03 (24.46–26.43)	20.57±1.67 (19.11-21-92)	20.45±1.68 (19.86–21.04)	22.32±1.92 (22.32–22.32)	20.08±1.46 (17.33–23.50)	22.17±2.02 (22.17–22.17)	23.68±2.07 (23.68–23.68)										nc
13. Beckerina (n = 1)	21.90±1.97 (21.90–21.90)	18.06±1.61 (17.87–18.25)	19.65±1.84 (19.65–19.65)	19.32±1.50 (18.38–21.94)	25.03±1.96 (23.64–26.42)	20.88±1.60 (19.67–23.62)	19.53±1.67 (19.14–19.92)	21.70±1.87 (21.70–21.70)	17.95±1.32 (15.56–21.79)	17.66±1.73 (17.66–17.66)	21.75±1.87 (21.75–21.75)	22.31±1.95 (22.31–22.31)									nc
14. Myriophora (n = 4)	20.34±1.52 (17.29–24.04)	16.51±1.20 (14.20–18.62)	19.04±1.46 (18.60–19.47)	17.36±1.19 (12.45–21.36)	23.49±1.71 (21.39–26.28)	18.11±1.27 (14.85–23.70)	17.66±1.33 (16.63–18.77)	21.92±1.61 (20.21–24.27)	16.58±1.04 (8.86–24.54)	18.37±1.43 (16.58–21.02)	22.87±1.67 (21.22–24.14)	20.00±1.60 (18.99–20.84)	18.34±1.50 (16.96–20.50								14.90±1.13 (13.02–18.64)
15. Borophaga (n = 2)	15.71±1.35 (15.35–16.06)	20.66±1.56 (18.93–23.26)	16.93±1.56 (16.62–17.23)	17.87±1.31 (16.78–19.93)	20.54±1.54 (18.63–21.79)	18.65±1.38 (17.47–20.40)	20.68±1.61 (19.88–21.58)	21.23±1.69 (20.20–22.26)	20.17±1.35 (16.95–23.09)	19.93±1.71 (18.91–20.95)	24.33±1.91 (24.23–24.42)	23.71±1.98 (23.31–24.11)	22.21±1.81 (22.11–22.30)	20.18±1.44 (17.51–22.74)							14.13
16. Chaetopleurophora (n = 1)	19.27±1.79 (19.27–19.27)	21.24±1.70 (21.14–21.34)	19.83±1.74 (19.83–19.83)	17.13±1.34 (15.88–18.55)	25.21±1.90 (24.40–26.01)	16.98±1.29 (14.83–17.82)	21.54±1.79 (21.37–21.71)	23.82±1.96 (23.82–23.82)	20.99±1.46 (18.37–24.59)	21.69±1.93 (21.69–21.69)	24.59±2.06 (24.59–24.59)	23.82±2.10 (23.82–23.82)	22.67±1.98 (22.67–22.67)	21. 68±1.58 (16.07–24.43)	19.94±1.60 (19.67–20.21)						nc
17. Apocephalus (n = 1)	21.40±1.89 (2140–21.40)	18.78±1.62 (17.60–19.96)	22.25±1.97 (22.25–22.25)	22.16±1.65 (19.90–25.64)	24.42±1.92 (24.06–24.79)	22.38±1.71 (21.17–23.94)	22.58±1.83 (22.28–22.89)	23.45±2.01 (23.45–23.45)	19.95±1.43 (17.01–23.89)	20.63±1.86 (20.63–20.63)	20.73±1.80 (20.73–20.73)	23.76±2.16 (23.76–23.76)	19.40±1.80 (19.40–19.40)	20.11±1.54 (16.58–21.02)	23.46±1.93 (23.34–23.58)	25.27±2.20 (25.27–25.27)					nc
18. Anevrina (n = 8)	16.78±1.41 (15.01–18.38)	19.25±1.49 (16.75–21.15)	16.21±1.32 (14.84–17.49)	15.29±1.08 (12.96–18.96)	21.14±1.65 (18.42–24.53)	15.53±1.12 (13.12–18.00)	18.88±1.42 (17.49–20.22)	20.00±1.54 (18.37–21.70)	18.48±1.18 (14.15–23.98)	16.57±1.30 (14.83–18.04)	23.30±1.74 (22.29–24.83)	21.39±1.70 (19.48–23.48)	20.07±1.52 (18.76–21.95)	19.50±1.34 (16.58–22.75)	18.09±1.36 (15.72–19.69)	19.52±1.48 (18.37–22.29)	24.53±1.81 (22.53–25.70)				11.02±0.89 (4.65–13.87)
19. Chaetogodavaria (n = 1)	15.00±1.57 (15.00–15.00)	17.93±1.52 (17.49–18.38)	15.40±1.56 (15.40–15.40)	16.65±1.35 (15.52–20.07)	21.96±1.84 (21.36–22.57)	16.61±1.34 (14.83–17.82)	19.05±1.71 (18.57–19.52)	18.03±1.67 (18.03–18.03)	17.76±1.27 (14.49–20.52)	16.76±1.73 (16.76–16.76)	21.56±1.82 (21.56–21.56)	23.87±2.15 (23.87–23.87)	18.59±1.75 (18.59–18.59)	18.86±1.46 (16.07–23.31)	16.17±1.39 (15.39–16.96)	20.20±1.80 (20.20–20.20)	22.59±1.92 (22.59–22.59)	16.44±1.41 (15.00–17.47)			nc
20. Stichillus (n = 2)	14.92±1.37 (14.84–1.47)	19.34±1.53 (17.83–20.59)	16.43±1.49 (16.25–16.60)	16.02±1.22 (14.32–18.24)	19.87±1.58 (18.05–21.36)	16.59±1.27 (14.83–17.83)	18.38±1.56 (17.65–19.10)	18.19±1.56 (18.18–18.19)	17.86±1.20 (14.31–21.54)	15.44±1.49 (15.18–15.70)	23.25±1.84 (22.69–23.82)	21.30±1.87 (21.05–21.55)	21.35±1.82 (20.57–22.13	19.12±1.42 (17.29–21.61)	14.45±1.23 (12.31–16.09)	20.02±1.62 (19.27–20.76)	22.07±1.82 (21.82–22.31)	16.01±1.29 (14.15–18.75)	12.80±1.28 (11.94–13.64)		8.06
21. Latiborophaga (n = 1)	17.85±1.70 (17.85–17.85)	22.13±1.75 (21.18–23.08)	19.50±1.95 (19.50–19.50)	20.44±1.57 (18.39–21.72)	24.53±1.87 (24.21–24.84)	21.91±1.69 (21.18–23.23)	24.05±1.89 (23.30–24.80)	21.32±1.81 (21.32–21.32)	21.57±1.50 (18.74–24.81)	20.60±1.91 (20.60–20.60)	22.65±1.88 (22.65–2265)	22.50±1.93 (22.50–22.50)	22.32±1.91 (22.32–22.32)	23.22±1.69 (21.53–24.24)	17.51±1.51 (16.79–18.23)	23.04±1.99 (23.04–23.04)	23.09±2.04 (23.09–23.09)	20.91±1.61 (20.05–22.51)	16.96±1.67 (16.96–16.96)	17.62±1.62 (17.55–17.69)	nc

nc: The values were not calculated due to due to the low sequence number.

**Table 4:** K2P genetic distances ± standard deviations (SD) for studied scuttle flies genera based on 481–549 bps of 28S rRNA sequences.

Group	Mean intergeneric genetic divergence ±SD (min–max)													mean intrageneric genetic divergence ±SD (min–max)
	1	2	3	4	5	6	7	8	9	10	11	12	13	
1. *Conicera* (n = 3)														0.42±0.23
2. *Gymnophora* (n = 2)	1.26±0.45 (1.05–1.69)													0.42±0.30
3. *Triphleba* (n = 1)	0.84±0.38 (0.63–1.26)	0.63±0.33 (0.42–0.84)												nc
4. *Dohrniphora* (n = 1)	3.07±0.79 (3.00–3.21)	3.22±0.83 (3.21–3.22)	3.22±0.83 (3.22–3.22)											nc
5. *Phora* (n = 2)	2.47±0.68 (2.33–2.54)	3.64±0.82 (3.63–3.64)	3.20±0.79 (3.20–3.20)	4.30±0.94 (4.30–4.30)										0
6. *Diplonevra* (n = 1)	1.76±0.57 (1.69–1.90)	1.91±0.60 (1.90–1.91)	1.91±0.61 (1.91–1.91)	2.12±0.66 (2.12–2.12)	3.86±0.88 (3.86–3.86)									nc
7. *Metopina* (n = 2)	1.27±0.48 (1.05–1.69)	0.21±0.15 (0.00–0.42)	0.42±0.28 (0.42–0.42)	3.22±0.84 (3.22–3.22)	3.64±0.83 (3.64–3.64)	1.91±0.61 (1.91–1.91)								0
8. *Mahabadphora* (n = 1)	3.57±0.78 (3.43–3.64)	4.31±0.88 (4.31–4.31)	3.86±0.85 (3.86–3.86)	4.98±1.01 (4.98–4.98)	2.77±0.72 (2.77–2.77)	4.53±0.94 (4.53–4.53)	4.31±0.89 (4.31–4.31)							nc
9. *Megaselia* (n = 62)	1.79±0.50 (1.48–3.88)	0.75±0.23 (0.00–2.55)	0.96±0.31 (0.42–2.56)	3.64±0.84 (3.21–4.78)	4.11±0.85 (3.64–5.88)	2.46±0.64 (1.91–4.10)	0.54±0.18 (0.00–2.12)	4.63±0.89 (4.31–5.66)						0.77±0.23
10. *Apodicrania* (n = 1)	1.27±0.48 (1.05–1.69)	0.21±0.15 (0.00–0.42)	0.42±0.28 (0.42–0.42)	3.22±0.84 (3.22–3.22)	3.64±0.83 (3.64–3.64)	1.91±0.61 (1.91–1.91)	0.00±0.00 (0.00–0.00)	4.31±0.89 (4.31–4.31)	0.54±0.18 (0.00–2.12)					nc
11. *Chaetogodavaria* (n = 1)	1.76±0.57 (1.69–1.90)	2.33±0.70 (2.33–2.33)	1.90±0.63 (1.90–1.90)	3.21±0.84 (3.21–3.21)	3.85±0.88 (3.85–3.85)	2.12±0.66 (2.12–2.12)	2.33±0.72 (2.33–2.33)	4.31±0.92 (4.31–4.31)	2.84±0.72 (2.33–4.54)	2.33±0.72 (2.33–2.33)				nc
12. *Anevrina* (n = 8)	0.81±0.37 (0.42–1.26)	0.63±0.33 (0.42–0.84)	0.03±0.03 (0.00–0.21)	3.19±0.83 (3.00–3.22)	3.17±0.79 (2.98–3.20)	1.88±0.60 (1.69–1.91)	0.44±0.29 (0.42–0.63)	3.84±0.84 (3.64–3.86)	0.99±0.32 (0.42–2.77)	0.44±0.29 (0.42–0.63)	1.88±0.62 (1.69–1.90)			0.05±0.05
13. *Borophaga* (n = 2)	1.05±0.41 (0.84–1.47)	1.47±0.54 (1.47–1.48)	1.05±0.46 (1.05–1.05)	3.00±0.79 (3.00–3.00)	3.41±0.81 (3.41–3.41)	2.12±0.65 (2.12–2.12)	1.48±0.56 (1.48–1.48)	3.86±0.87 (3.86–3.86)	2.01±0.58 (1.47–3.65)	1.48±0.56 (1.48–1.48)	1.05±0.46 (1.05–1.05)	1.02±0.45 (0.84–1.05)		0
14. *Stichillus* (n = 1)	1.97±0.61 (1.90–2.12)	2.99±0.77 (2.98–2.99)	2.55±0.72 (2.55–2.55)	3.21±0.77 (3.21–3.21)	4.07±0.88 (4.07–4.07)	2.77±0.75 (2.77–2.77)	2.99±0.79 (2.99–2.99)	4.08±0.87 (4.08–4.08)	3.33±0.76 (2.98–4.31)	2.99±0.79 (2.99–2.99)	1.69±0.59 (1.69–1.69)	2.52±0.71 (2.33–2.55)	1.48±0.54 (1.48–1.48)	nc

nc: The values were not calculated due to due to low sequence number.

**Table 5:** K2P genetic distances ± standard deviations (SD) for studied scuttle flies genera based on 543 bps of *Arginine kinase* sequences.

Group	Mean intergeneric genetic divergence ±SD (min–max)									mean intrageneric genetic divergence ±SD (min–max)
	1	2	3	4	5	6	7	8	9	
1. *Mahabadphora* (n = 1)										nc
2. *Myriophora* (n = 4)	11.69±1.38 (10.59–13.90)									4.16±0.61
3. *Megaselia* (n = 1)	12.75±1.57 (12.75–12.75)	8.02±1.09 (7.65–8.72)								nc
4. *Apocephalus* (n = 2)	14.91±1.59 (14.34–15.48)	10.47±1.19 (9.55–11.29)	9.63±1.19 (9.31–9.95)							5.41±0.97
5. *Apodicrania* (n = 1)	14.32±1.68 (14.32–14.32)	8.80±1.16 (8.25–9.34)	9.12±1.33 (9.12–9.12)	11.07±1.42 (10.21–11.94)						nc
6. *Gymnophora* (n = 1)	11.64±1.50 (11.64–11.64)	8.38±1.14 (7.65–9.32)	9.73±1.39 (9.73–9.73)	10.75±1.33 (10.66–10.83)	10.41±1.46 (10.41–10.41)					nc
7. *Phalacrotophora* (n = 1)	15.99±1.81 (15.99–15.99)	10.14±1.29 (9.54–10.82)	12.60±1.58 (12.60–12.60)	14.09±1.67 (13.53–14.65)	12.19±1.54 (12.19–12.19)	11.27±1.48 (11.27–11.27)				nc
8. *Melaloncha* (n = 1)	15.22±1.78 (15.22–15.22)	11.87±1.40 (11.05–13.59)	10.22±1.41 (10.22–10.22)	11.99±1.43 (11.31–12.66)	10.46±1.51 (10.46–10.46)	12.42±1.61 (12.42–12.42)	14.26±1.64 (14.26–14.26)			nc
9. *Beckerina* (n = 1)	14.53±1.64 (14.53–14.53)	13.52±1.53 (12.09–15.04)	14.61±1.65 (14.61–14.61)	15.43±1.66 (15.28–15.59)	12.85±1.64 (12.85–12.85)	11.66±1.46 (11.66–11.66)	17.03±1.87 (17.03–17.03)	16.07±1.81 (16.07–16.07)		nc
10. *Kerophora* (n = 1)	13.39±1.61 (13.39–13.39)	8.04±1.08 (7.62–9.08)	9.08±1.31 (9.08–9.08)	10.89±1.42 (10.35–11.43)	10.83±1.44 (10.83–10.83)	9.51±1.34 (9.51–9.51)	7.66±1.16 (7.66–7.66)	11.04±1.40 (11.04–11.04)	14.85±1.71 (14.85–14.85)	nc

nc: The values were not calculated due to due to low sequence number.

**Table 6:** Sequence divergences (K2P) at the three loci for scuttle flies at interspecific/intergeneric levels.

Loci	Indices	Number of taxa analyzed	Range (%)	Mean distance (%)
*COI*	IPD	71 spp. belonged to 9 genera	0–27.26	16.67
	IGD	117 spp. belonged to 21 genera	12.80–25.44	17.30
*28S*	IPD	71 spp. belonged to 9 genera	0–6.24	1.39
	IGD	88 spp. belonged to 14 genera	0–4.98	1.29
*AK*	IPD	14 spp. belonged to 10 genera	2.27–17.03	10.76
	IGD	14 spp. belonged to 10 genera	7.66–17.03	10.76

IPD: Interspecific pairwise distance, IGD: Intergeneric genetic distances.

#### Relationship analysis

**Fig 4:**
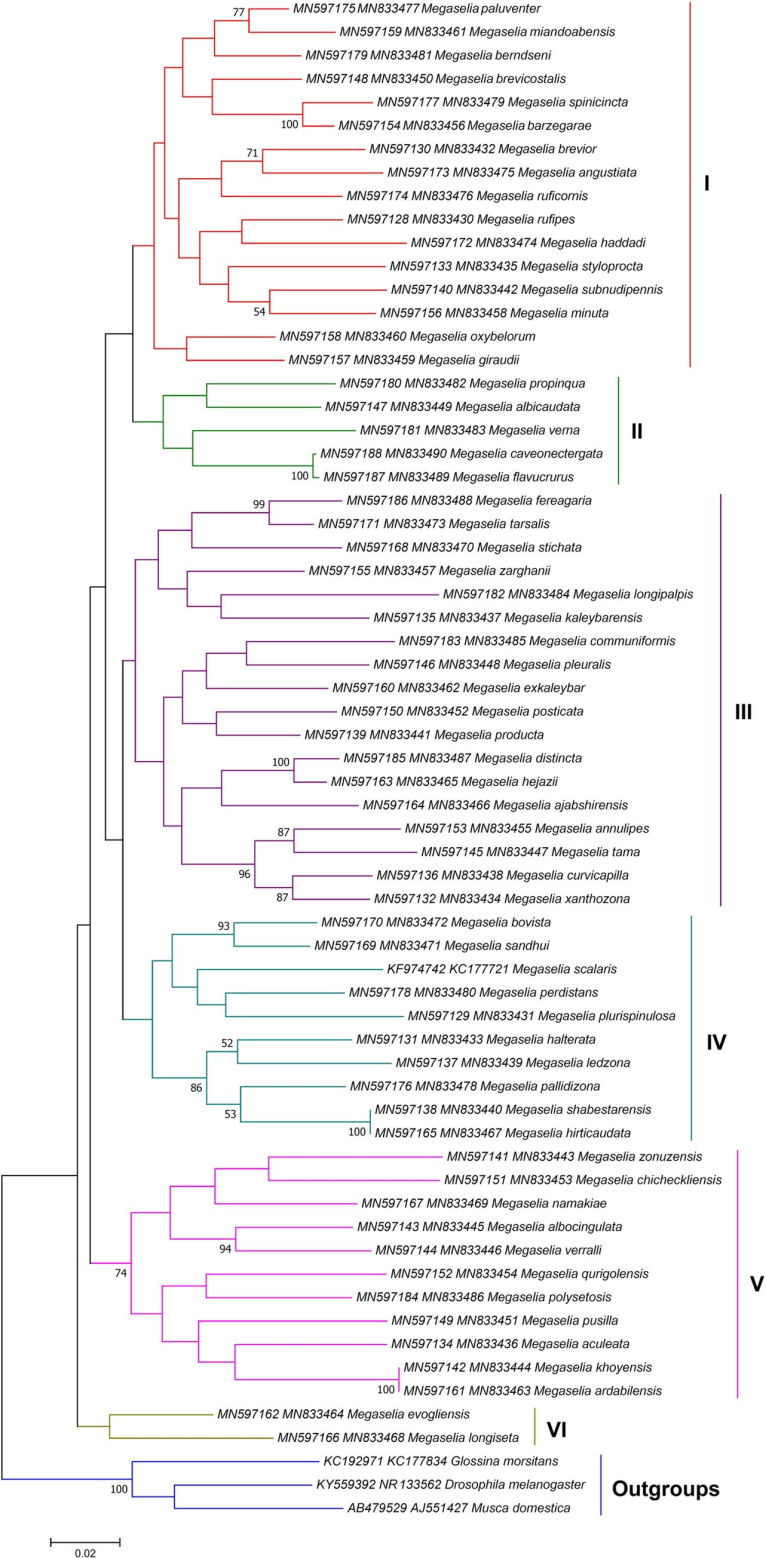
Maximum likelihood tree inferred from 1200–1269 bp of the *COI*-*28S* gene sequences of 61 *Megaselia* species obtained in this study, along with the sequences of *M*. *scalaris* from the Genbank (KF974742-KC177721). Only bootstrap values higher than 50% are shown on the branches. The bar indicates substitutions per site. The *Drosophila melanogaster* (Meigen, 1830) (KY559392-NR_133562), *Glossina morsitans* Westwood, 1851 (KC192971**-**KC177834) and *Musca domestica* Linnaeus, 1758 (AB479529**-**AJ551427) were set as outgroups.

**Fig 5:**
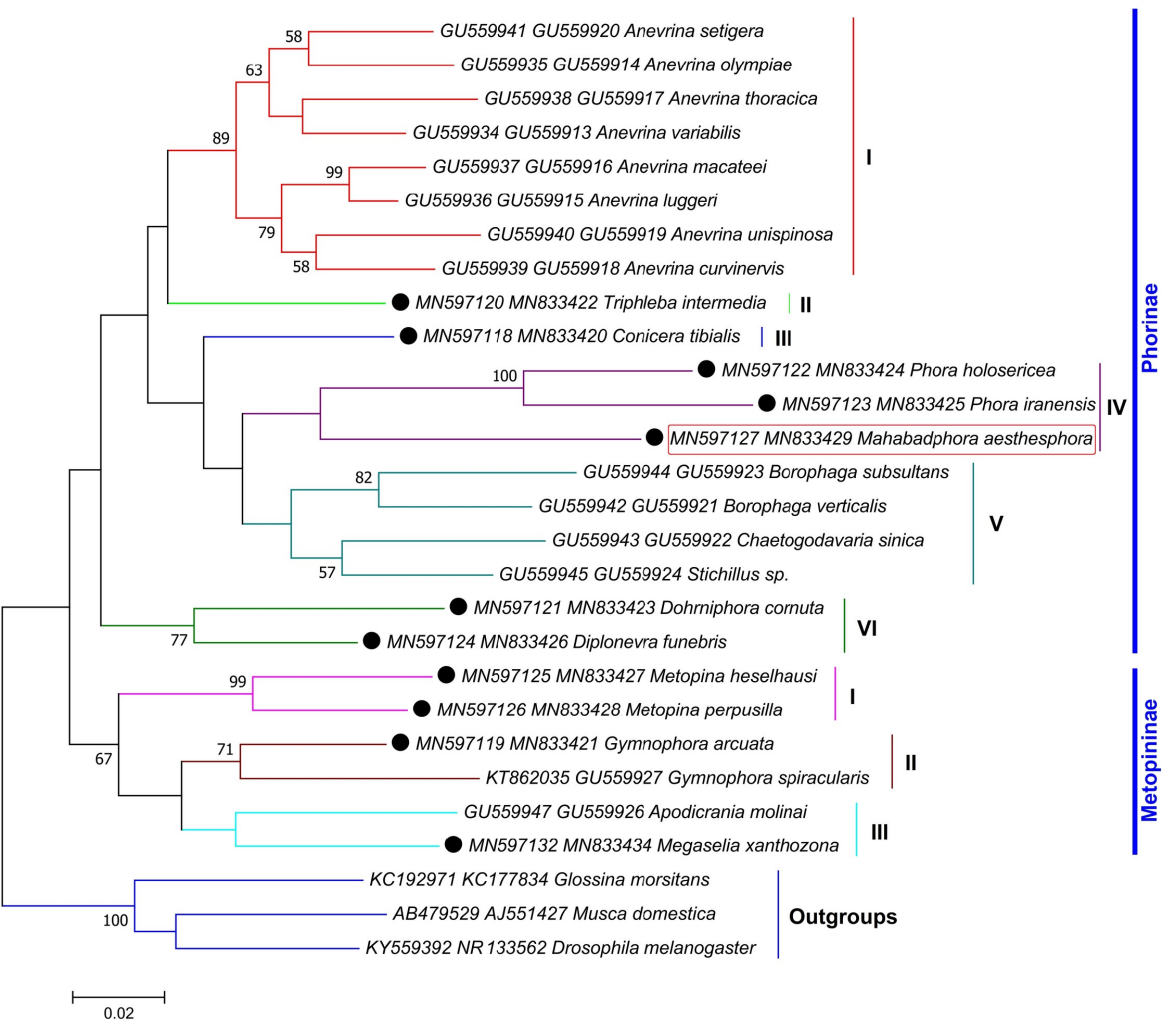
Maximum likelihood tree inferred from 1200–1269 bp of the *COI*-*28S* gene sequences of 11 non-*Megaselia* species obtained in this study. Only bootstrap values higher than 50% are shown on the branches. The bar indicates substitutions per site. The *Drosophila melanogaster* (Meigen, 1830) (KY559392 and NR_133562), *Glossina morsitans* Westwood, 1851 (KC192971**-**KC177834) and *Musca domestica* Linnaeus, 1758 (AB479529**-**AJ551427) were set as outgroups.

**Fig 6:**
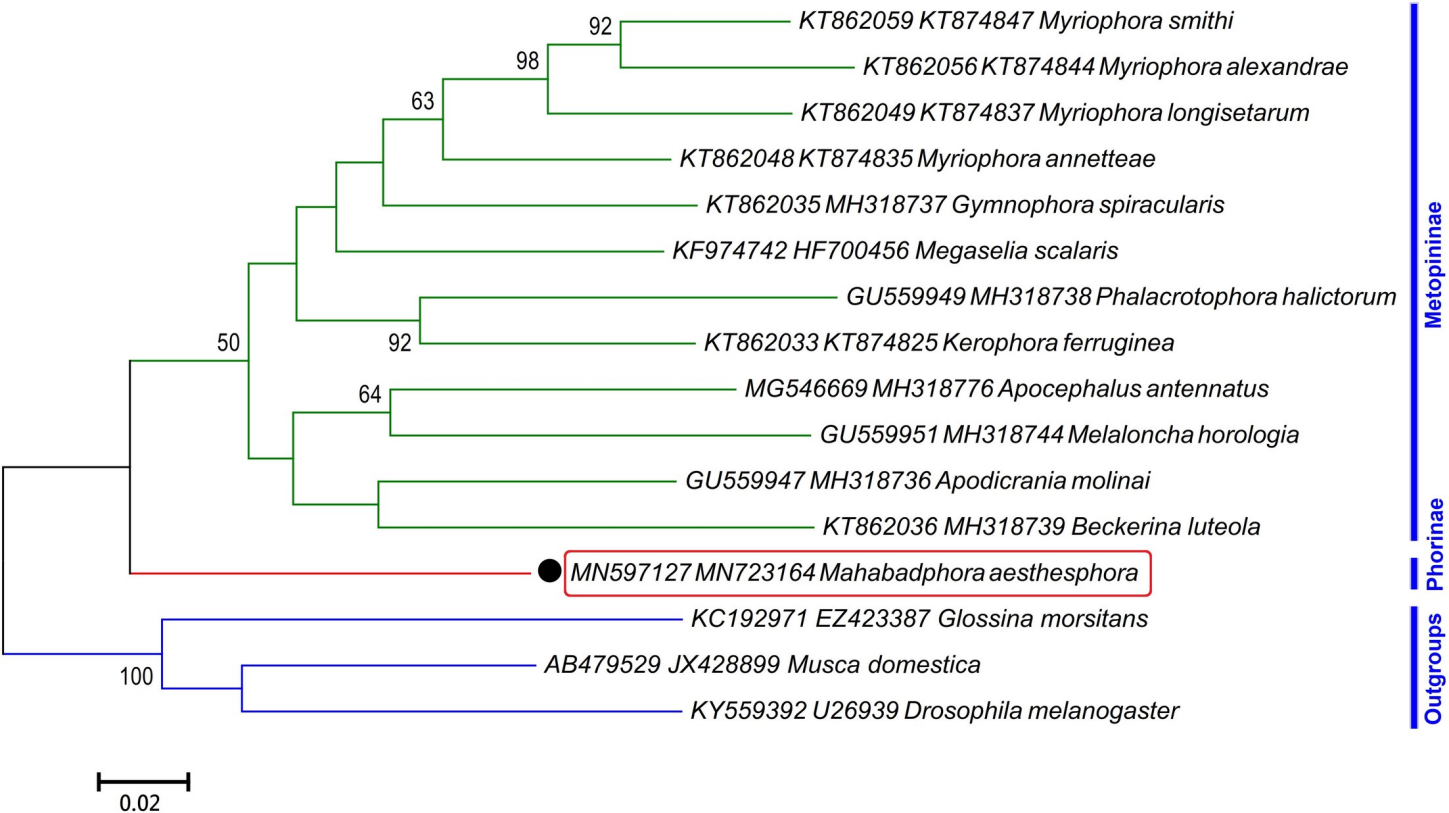
Maximum likelihood tree inferred from 1263 bp of the *COI*-*AK* gene sequences of the new genus described in this study. Only bootstrap values higher than 50% are shown on the branches. The bar indicates substitutions per site. The *Drosophila melanogaster* (Meigen, 1830) (KY559392 and U26939), *Glossina morsitans* Westwood, 1851 (KC192971**-**EZ423387) and *Musca domestica* Linnaeus, 1758 (AB479529**-**JX428899) were set as outgroups.

The ML method provided more reliable phylogenetic trees than NJ method (S1–S3 Figs). Consequently, three ML phylogenetic trees were constructed using combined sequences of *COI*-*28S* (1200–1269 bp) and *COI*-*AK* (1263 bp) datasets (Figs 4–6). The first one was drawn based on *COI*-*28S* dataset for the *Megaselia* spp. determined in this study along with the sequences of *M*. *scalaris* (KF974742-KC177721), *D*. *melanogaster* (KY559392-NR133562), *Glossina morsitans* (KC192971-KC177834) and *Musca domestica* (AB479529-AJ551427) from the Genbank (Fig 4). The tree divided the *Megaselia* species into six groups. Sixteen similar morphospecies clustered together in the clade I. Morphological parsing showed that in all species grouped in this clade, except for *M*. *styloprocta*, the mesopleuron was bare, and dorsal face of epandrium was longer than or equal to the length of the anal tube. The *M*. *styloprocta* was joined to *M*. *minuta* and *M*. *subnudipennis* branch as a sister group. Our study found four out of the 10 members of the *M*. *brevior* complex, all of which were correctly classified in the clade I.

Clade II was composed of five species: *M*. *albicaudata*, *M*. *propinqua*, *M*. *verna*, *M*. *flavucrurus*, and *M*. *caveonectergata*, which shared many morphological similarities. There were 18 species categorized in Clade III, where all species, except *M*. *longipalpis*, *M*. *fereagaria* and *M*. *tarsalis*, had hairy mesopleuron. *M*. *longipalpis* could be distinguished from other species by having large palps and the latter two species varied slightly from eachother in the color of the legs and the shape of the hypopygium.

Clade IV included 10 similar morphospecies and was divided into two subclades. In the first clade *M*. *sandhui* / *M*. *bovista*, two morphologically very similar species were clustered with *M*. *scalaris*, *M*. *perdistans* and *M*. *plurispinulosa*. The second clade comprised of two members of *M*. *sulphuripes* species group (*M*. *halterata* and *M*. *hirticaudata*) together with three other species. *M*. *shabestarensis* and *M*. *ledzona* were new species that were presented to the world fauna by our research team; however, the former species was molecularly indistinguishable from *M*. *hirticaudata*.

In clade V, there were 11 species that shared similar morphological properties, e.g. the hairs of left side of epandrium were, at most, only as robust as those of cerci. *Megaselia ardabilensis* was morphologically very similar to *M*. *khoyensis* but could be distinguished by the relative size of hairs below basal half of hind femur, as compared to those of anteroventral row of outer half. Moreover, their hypandrial lobes were clearly different.

The clade VI comprising of *M*. *evogliensis* / *M*. *longiseta* was established as paraphilitic group of other megaselias in the phylogenetic tree. The *M*. *evogliensis* and *M*. *longiseta* species had definite dissimilarities (see the key).

The second tree covered the *COI-28S* sequences of other 11 species found in this study, along with those of 17 other species retrieved from the Genbank (Fig 5). The tree divided the studied taxa into two subfamilies: Phorinae (six clades) and Metopininae (three clades). Taxonomically challenging species *Triphleba intermedia* and *Conicera tibialis* were arranged in clades II and III of Phorinae, respectively. The newly described taxon, *Mahabadphora aesthesphora*, and the genus *Phora* were classified in the clade IV under the Phorinae subfamily. They shared some morphological characteristics e.g. vein Rs without hairs along the dorsal face, at least two differentiated dorsal or near-dorsal bristles in basal two-thirds of mid tibia and hind tibia without longitudinal hair palisade. However, unlike *Phora*, the Vein 3 in *Mahabadphora*, was forked. The *Diplonevra* and *Dohrniphora* genera were organized in the same expected clade (VI) as a sister group of other species of subfamily Phorinae.

Four phorid fly species of this study (*Metopina heselhausi*, *Metopina perpusilla*, *Gymnophora arcuata* and *Megaselia xanthozona*), along with a couple species from the GenBank (*Gymnophora spiracularis* (KT862035, GU559927) and *Apodicrania molinai* (GU559947, GU559926)), yielded three strongly supported and genetically quite distant clades within Metopininae subfamily.

The third ML consensus tree, in support of the second one, recovered from 16 pairs of *COI-AK* sequences confirmed that *Mahabadphora aesthesphora* was a genetically quite distant clade from Metopininae, clearly representing valid taxon in the Phorinae scuttle flies (Fig 6).

#### Checklist of scuttle flies occurring in Iran and their identification key

**Fig 7:**
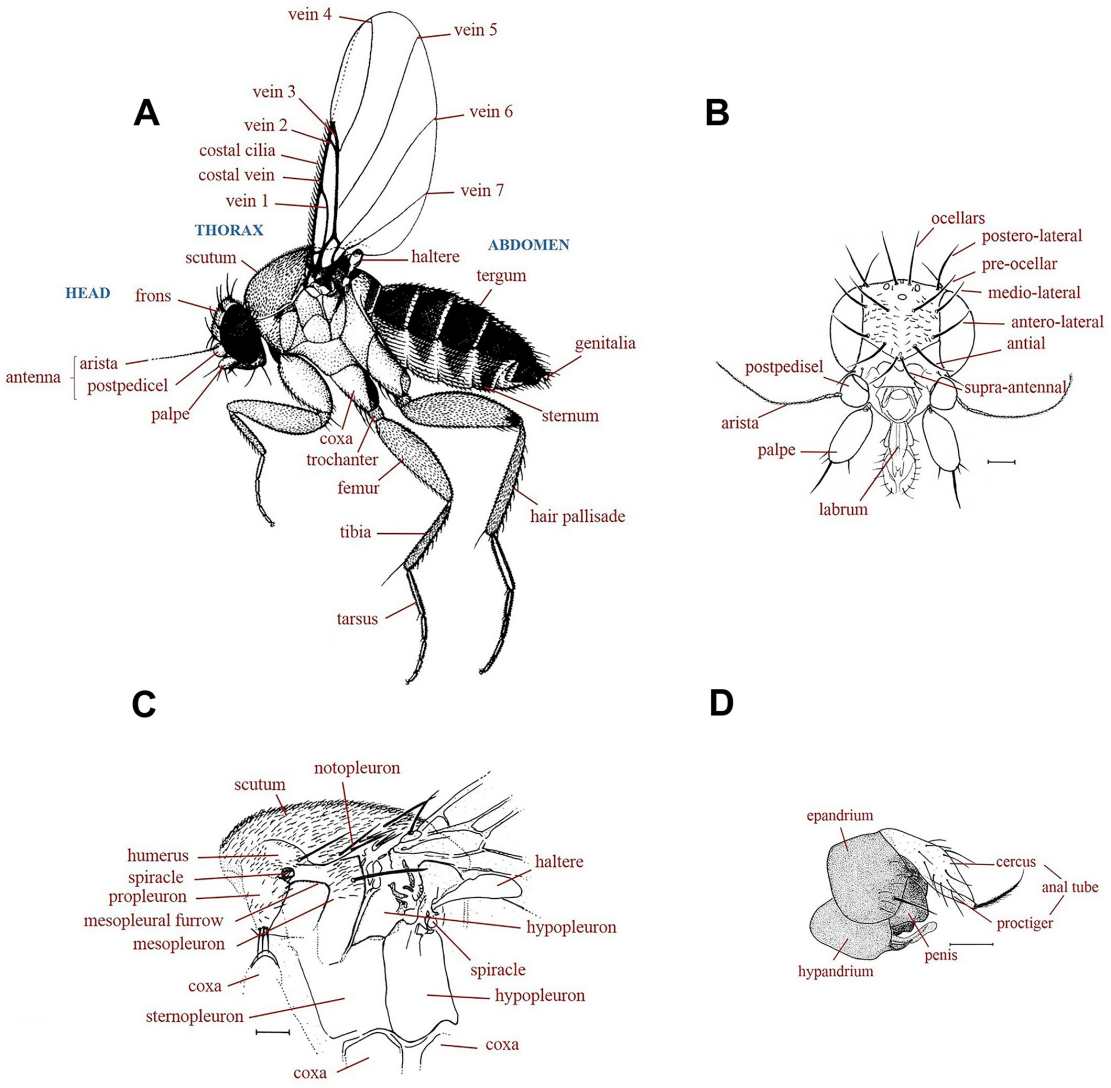
Characters of adult male phorid flies used in the key. A, Lateral view of whole fly (*Megaselia scalaris*); B, frontal view of head (*Triphleba nudipalpis*), showing bristles; C, left side of thorax (*Megaselia ciliata*); D, left side view of male hypopygium (*Megaselia scalaris*). Modified from Smith, 1986.

An inventory of 97 scuttle fly species known from Iran, together with their collection data, hierarchical classification, and synopsis of their life history is summarized in Table 2. These flies were distributed to several locations of 12 Provinces of the country, namely Alborz, Ardabil, East Azerbaijan, Fars, Golestan, Kermanshah, Markazi, Mazandaran, Razavi Khorasan, Tehran, West Azerbaijan, and Zanjan. They are organized into three subfamilies of Chonocephalinae, Metopininae, and Phorinae and 13 genera. Moreover, 18 species in seven genera *Arabiphora* Disney, 2006, *Chonocephalus* Wandolleck, 1898, *Diplonevra* Lioy, 1864, *Megaselia* Rondani, 1856, *Metopina* Macquart, 1835, *Phalacrotophora* Enderlein, 1912 and *Phora* Latreille, 1796 had formerly been reported from Iran [49,65,69,79,82,100]. The following key is based on the 87 adult male species of scuttle flies described in this project and on 10 species described previously. The key characters are illustrated in Fig 7.

### Key to the genera and species of the Phoridae known from Iran

The following key is based on the male insects of phorid spp. described during this project and on 10 species published previously [1,2,60,63,64,70,79,99,103–111].

* Identifications should be confirmed by reference to the description and figures in the relevant publication.

1. Apex of third antennal segment drawn out into long …………………………………………………2
  - Apex of third antennal segment not drawn out in this way……………………………………….…….7
2. Mid tibia with only one dorsal bristle in basal half (genus *Conicera*) ……………………………… 3
  - Mid tibia without such bristles………………………………………………………………………… 4
3. Claspers of hypopygium developed as irregular lobes, which are not tapered and tend to be rounded, posterior face of mid femur with a sense organ and pit of that larger and apical process longer…………………………………………………………………………………*Conicera tibialis*
  - Without this combination…………………………………………………………………… other species
4. Costal index more than 0.5 (genus *Iranphora*) ……………………………………………………… 5
  - Costal index between 0.4 and 0.5 (genus *Arabiphora*) ……………………………………………… 6
5. Notopleuron with three bristles. Mesopleuron bare. Abdominal tergites with small hairs largely restricted to hind margins. Venter pale and without hairs. Hypopygium pale with the pale anal tube that is longer than epandrium. Wings 0.87 mm long. Costal index 0.64. Haltere knob brown……………………………………………………………………… *Iranphora sharafkhaneensis*
  - Without this combination…………………………………………………………………… other species
6. Frons about 3x as broad as midline length; brown, with darker ocellar triangle; 12‑16 hairs. Postpedicels pale yellowish brown, lacking SPS vesicles, Wing 1.2‑1.3 mm long. Costal index 0.47…………………………………………………………………………. *Arabiphora tenuifemorata*
  - Without this combination…………………………………………………………………… other species
7. Vein 6 (third thin vein) with a sudden bend near middle, opposite (and opposed to) basal curve of vein 5 (genus *Metopina*)… ……………………………………………………………………………… 8
  - Vein 6 without such a bend…………………………………………………………………………… 10
8. Posterior face of base of hind femur with conspicuous "sensory patch" that includes a distinct circular pit. Hairs of hind trochanter reduced in number and with only a single, basal one evident on ventral face, which is itself fringed with dense microscopic hairs. In addition, the terminal spine is dark, somewhat reduced and strongly tapered…………………………………………. *Metopina heselhausi*
  - Sensory patch of hind femur less conspicuous and without a pit. At least three hairs along ventral face of hind trochanter, which is itself fringed with sparse, inconspicuous microscopic hairs. The terminal spine is more robust, pale, and more gradually tapered…………………………………………… 9
9. Venter with a distinct ventral plate on segment 4, bearing irregular rows of hairs along the lateral margins but none along the median third, sensory patch on posterior face of base of hind femur usually visible as a darker smudge at relatively low magnification…………………… … *Metopina oligoneura*
  - Venter without a clearly defined plate on segment 4 and along with hair patch usually more than 20 hairs, microsculpture of posterior face of hind femur with rows of polygons curving dorsally…………………………………………………………………………… *Metopina perpusilla*
10. Hind tibia simply haired on dorsal face, but may bear isolated bristles…………………….………. 11
  - Hind tibia with one or more dorsal or near dorsal longitudinal palisade-like rows of setae………… 20
11. Middle and hind tibiae with isolated bristles………………………………………………………. 12
  - Middle and hind tibiae without isolated bristles………………………………………………….… 17
12. Middle tibia with at most one dorsal bristle (genus *Triphleba*) …………………………………… 13
  - Middle tibia with at least two dorsal bristles …………………………………………………………… 14
13. Scutellum with two pairs of bristles, both of which are clearly more robust and longer than hairs of scutum, left side of epandrium with process divided into two arms, lower arm of process of left side of epandrium very much larger than upper arm, hind tibia usually with a bristle in upper half on anterior face ……………………………………………………………………… *Triphleba intermedia*
  - Without this combination…………………………………………………………………… other species
14. Vein 3 unforked (genus *Phora*) ……………………………………………………………………. 15
  - Vein 3 forked (genus *Mahabadphora*) ……………………………………………………………… 16
15. Left side of epandrium deeply cleft to give a shorter upper lobe and a longer lower lobe, upper lobe of left side of epandrium with a nearly vertical posterior margin, which is irregularly straight-edged to somewhat concave in middle, and is distinctly crenellate ………………………… *Phora holosericea*
  - Left side of the epandrium not being deeply cleft, upper lobe of left side of epandrium otherwise……………………………………………………………………………… *Phora iranensis*
16. Scutellum with a pair of long bristles and a pair of short hairs. The hairs of the tergites being very small and the venter lacking hairs. Epandrium with strong bristles, left lobe of the hypandrium smaller than right lobe, wing length 1.44 mm, costal index 0.31 ……………… *Mahabadphora aesthesphora*
  - Without this combination…………………………………………………………………… other species
17. Frons lacking bristles between antennae and ocelli (genus *Gymnophora*) ………………………. 18
  - Frons with bristles between antennae and ocelli (genus *Chonocephalus*) …………………………… 19
18. Oblique ridge of notopleuron largely pale. Small swelling of costa before tip of vein 1 embracing a pale oval spot …………………………………………………………………. *Gymnophora arcuata*
  - Without this combination…………………………………………………………………… other species
19. Microsetae of left side of epandrium near margin absent, epandrium with numerous long hairs, right gonopod asymmetrically expanded distally and with the longer arm directed rearwards; left gonopod a long narrow process bearing a pair of bristles at its slightly expanded tip……………………………………………………………………………… *Chonocephalus heymonsi*
  - Without this combination…………………………………………………………………… other species
20. Hind tibiae with two (rarely three) setal palisades (genus *Diplonevra*) ……………………………. 21
  - Hind tibia with only one dorsal palisade-like row of setae ………………………………………… 22
21. Halteres black, proboscis elongated, narrow, and elbowed. Hind trochanter with cluster of short black ’studs’ in place of bristles …………………………………………… *Diplonevra funebris*
  - Without this combination…………………………………………………………………… other species
22. Middle tibia with two isolated bristles in proximal third (genus *Dohrniphora*) …………………… 23
  - Middle tibia without isolated bristles in proximal thirds ……………………………………………… 24
23. Legs yellow apart from some brown pigment on mid coxae, posterior face of hind femur with sparse dorsal setae; ventrobasal region with only four or five short, thick peg-like setae……………………………………………………………………………… *Dohrniphora cornuta*
  - Without this combination…………………………………………………………………… other species
24. Male with proctiger ending in finely feathered bristles that are clearly more robust than setae on cerci, hind tibia with a clearly differentiated longitudinal row of stout, spine-like, antero-dorsal hair (genus *Phalacrotophora*) …………………………………………………………………………………… 25
  - Male with proctiger ending in setae that little, if any, stronger than those on cerci, hind tibia without a clearly differentiated row of spine-like antero-dorsal hairs (genus *Megaselia*) ……………………. 26
25. Wing about 2 mm long, costal index of 0.46 …………………………… *Phalacrotophora fasciata*
  - Wing 1.39 mm long, costal index 0.36 ……………………………………… *Phalacrotophora flavidus*
  - Wing much longer and costal index exceeds 0.5 ……………………………………………………….26
26. Abdominal spiracles greatly enlarged on segments 5–7 at least ………………. *Megaselia stigmatica*
  - Abdominal spiracles not enlarged …………………………………………………………………….27
27. Hairs at tip of anal tube developed as robust feathered bristles ………………… *Megaselia scalaris*
  - Hairs at tip of anal tube normal …………………………………………………… .………………… 28
28. Longest bristle on palp at most as long as maximum width of palp …………… *Megaselia longipalpis*
  - Longest bristle on palp clearly longer than maximum width of palp …………………………………29
29. Hypopygium is often largely straw yellow. The inner face of the epandrium comprises elaborate cavities lined with fine pale hairs ………………………………….……….…. *Megaselia xanthozona*
  - Hypopygium otherwise ………………………………………………………………………………. 30
30. Tibia and metatarsus of fore legs dark brown in apical half …………………… *Megaselia annulipes*
  - Tibia and metatarsus of fore legs uniformly yellowish or uniformly dark …………………………… 31
31. The apical third of hind tibia appears deformed, associated with the strong deflection of the hair palisade onto the anterior face ………………………………………………… *Megaselia hirtiventris*
  - The apical third of hind tibia not modified in this way ………………………………………………. 32
32. Midline length of dorsal face of epandrium very short and cerci lengthened dorsoventrally………………………………………………………………………… *Megaselia tama*
  - Hypopygium otherwise ………………………………………………………………………………. 33
33. Right side of the epandrium distinctively, dark hook-shaped posteroventral process………………………………………………………………………… *Megaselia albocingulata*
  - Hypopygium otherwise ………………………………………………………………………………. 34
34. Mesopleuron with hairs and sometimes with bristles …………………………………………. 35
  - Mesopleuron bare ……………………………………………………………………………………. 66
35. Mid femur with a conspicuous ventral dilation …………………………………………………… 36
  - Mid femur with no ventral dilation ………………………………………………………………… 37
36. Haltere knob pale …………………………………………………………… *Megaselia zonuzensis*
  - Haltere knob brown…………………………………………………………… *Megaselia qurigolensis*
37. Terminal hairs of anal tube weak and down-curved …………………………… *Megaselia producta*
  - Terminal hairs of anal tube often relatively strong and always curved upwards ……………… .…… 38
38. Mesopleuron with at least one bristle near posterior border. These bristles are clearly more robust and longer than hairs …………………………………………………………………………………… 39
  - Mesopleuron with hairs only. When these hairs are somewhat strong and bristle-like, there is no clear division into two size classes …………………………………………………………………………53
39. Scutellum with two pairs of bristles……………………………………………. *Megaselia daemon*
  - Scutellum with an anterior pair of short hairs and a posterior pair of long bristles…………………….40
40. Knob of haltere largely or entirely yellow …………………………………………………………. 41
  - Knob of haltere somewhat darkened ………………………………………………………………….43
41. Hairs below basal half of hind femur relatively short and in a single row ……… *Megaselia pleuralis*
  - Hairs below basal half of hind femur relatively long and somewhat crowded………………………… 42
42. Left side of epandrium with a strong bristle towards lower margin near half-way point and a vertical row or 2–4 slightly weaker bristles in front ……………………………………… *Megaselia stichata*
  - Left side of epandrium without this isolated bristle and vertical row of 3–4 stronger bristles usually less inclined anteriorly ……………………………………………………… *Megaselia meconicera*
43. Penis complex in hypopygium including a pale process tipped with a pair of short but thick spines………………………………………………………………………. *Megaselia chicheckliensis*
  - Penis complex in hypopygium otherwise …………………………………………………………… 44
44. Abdominal venter with hairs restricted to segments 5 and 6 ……………………………………… 45
  - Abdominal venter with hairs on segments 3–6 ……………………………………………………… 46
45. Venter of abdominal segment 6 with a posterior pair of bristle clusters, with each bristle arising from a dark circular disc ………………………………………………………………… *Megaselia aculeata*
  - Venter of abdominal segment 6 without such a bristle ……………………. *Megaselia ghalateshahensis*
46. Front basitarsus ventrally with hairs replaced by minute spinules …………………………………. 47
  - Front basitarsus ventrally without such hairs ………………………………………………………… 49
47. Hind tibia with six of the eight differentiated posterodorsal hairs robust …… *Megaselia evogliensis*
  - Hind tibia with more moderately differentiated posterodorsal hairs …………………………………. 48
48. The two hairs of the ventral edge of left cercus situated postero-ventrally so as to be almost as far back as lower hair of posterior edge of cercus. The most posterior of these ventral hairs is at least as strong as terminal hairs of proctiger, and usually stronger ……………………. *Megaselia curvicapilla*
  - Neither hair of cercus as strongly developed as terminal hairs of proctiger … *Megaselia mahabadensis*
49. Hypopygium with very long left hypandrial lobe ………………………………………………… 50
  - Not so ………………………………………………………………………………………………… 51
50. Hairs below basal half of hind femur clearly longer than those of anteroventral row of outer half………………………………………………………………………………… *Megaselia khoyensis*
  - Hairs below basal half of hind about as long as those of anteroventral row of outer half……………………………………………………………………………. *Megaselia ardabilensis*
51. Legs yellow ………………………………………………………………………… *Megaselia yaseri*
  - Legs brown to brownish yellow …………………………………………………………………… 52
52. Hairs below basal half of hind femur clearly longer than those of anteroventral half………………………………………………………………………………… *Megaselia pereensis*
  - Hairs below basal half of hind about as long as those of anteroventral row of outer half……………………………………………………………………………… *Megaselia namakiae*
53. The lower margin of right side of epandrium greatly extended downwards and curving under the hairless hypandrium ……………………………………………………………… *Megaselia verralli*
  - Epandrium not modified in this way ………………………………………………………………… 54
54. Ventral face of metatarsus of front leg with at least two complete longitudinal rows of hairs reduced to short blunt spines ………………………………………………………………………………… 55
  - Ventral face of fore metatarsus at most with only one complete row of hairs modified in this way…………………………………………………………………………………………………… 57
55. Notopleuron with two bristles …………………………………………………… *Megaselia zarghanii*
  - Notopleuron with three bristles ……………………………………………………………………… 56
56. Haltere knob brown ………………………………………………………… *Megaselia ajabshirensis*
  - Haltere knob whitish yellow ……………………………………………………… *Megaselia hejazii*
57. Labella of proboscis somewhat to conspicuously enlarged and their lower faces with dense fields of short, blunt, pale spine ……………………………………………………………………………… 58
  - Labella little, if any, enlarged and lower faces with few or no short pale spines ……………………… 60
58. Haltere knob yellow ……………………………………………………………… *Megaselia altifrons*
  - Haltere knob brown ………………………………………………………………………………… 59
59. Anterior pair of bristles on scutellum clearly shorter and finer than posterior pair. Apart from the lowest bristle, the left side of epandrium with weaker hairs ……………………… *Megaselia posticata*
  - Bristles on scutellum subequal, the anterior pair being only slightly shorter and finer. All hairs on left side of epandrium, in anterior half, are stronger ………………………… *Megaselia communiformis*
60. Anal tube clearly longer than the length of dorsal face of epandrium …………. *Megaselia styloprocta*
  - Anal tube subequal or clearly shorter than the length of dorsal face of epandrium ……………… 61
61. Haltere with stem and knob largely dark …………………………………………………………… 62
  - Haltere with knob mainly yellow or yellowish …………………………………………………… 64
62. Hairs of tergite of abdominal segment 6 strongly developed ………………… *Megaselia exkaleybar*
  - These hairs much finer ………………………………………………………………………………. 63
63. All legs dark grey to blackish …………………………………………………… *Megaselia pusilla*
  - Fore legs and mid legs yellow, hind legs yellow brown …………………………. *Megaselia polysetosis*
64. Hairs on left side of epandrium distinctly somewhat more robust than those on cerci……………………………………………………………………………. *Megaselia subpleuralis*
  - Hairs on left side of epandrium at most only as robust as those on cerci …………………………… 65
65. Hairs below basal half of hind femur clearly longer than those of anteroventral half……………………………………………………………………………. *Megaselia kaleybarensis*
  - Hairs below basal half of hind about as long as those of anteroventral row of outer half………………………………………………………………………………… *Megaselia distincta*
66. Abdominal tergites 1–6 with numerous long bristles and epandrium also with bristle……………………………………………………………………………… *Megaselia rufipes*
  - Any long bristles on abdominal tergites restricted to hind margins of 5 and 6 and sides of tergite 2.… 67
67. A short row of 4–5 spines (with bent tips) sharply contrasting with rest of hairs beneath base of hind femur………………………………………………………………………… *Megaselia longiseta*
  - No such spines beneath hind femur, but with hairs only …………………………………………… 68
68. Rear margin of segment 4 of venter forms a median ‘pocket’ associated with 2‑4 more robust hairs at sharply defined hind margin of sternum; and segment 5 with a pair of diverging ridges running rearwards from just behind these hairs ……………………………………………. *Megaselia sandhui*
  - Ventral face of segments of abdomen not in this form ……………………………………………… 69
69. Scutellum with two pairs of bristles ………………………………………………………… …… 70
  - Scutellum with a posterior pair of bristles and an anterior pair of hairs ………………………………72
70. Hind tibia with an antero-dorsal row of short black spines, clearly differentiated from adjacent hairs of anterior face, as well as the longer postero-dorsals (the dorsal hair palisade passes between these two rows of spines); mid tibia also with a row of differentiated antero-dorsals in addition to postero-dorsals ……………………………………………………………………… *Megaselia plurispinulosa*
  - Neither hind nor mid tibia with such spine-like antero-dorsals ……………………………………….71
71. Vein Sc strong and its tip fused to vein 1 (R1) …………………………………. *Megaselia ruficornis*
  - Vein Sc fades away before reaching vein 1 (R1) ……………………………………. *Megaselia giraudii*
72. Left side of epandrium with at least one bristle or strong hair, which is more robust than hairs of cerci………………………………………………………………………………………………… 73
  - Hairs of left side of epandrium at most only as robust as hairs of cerci, usually weaker ……………… 80
73. Bristles at rear margin of abdominal tergite 6 conspicuously longer and stronger than most robust hairs or bristles on left side of epandrium, and the hairs of the venter of segment 6 also strong and bristle-like ……………………………………………………………………… *Megaselia spinicincta*
  - Bristles at rear margin of abdominal tergite 6 subequal to or shorter than most robust hairs or bristles on left side of epandrium, and those on venter of segment 6, usually weaker ………………………… 74
74. Strong bristles on epandrium distinctly feathered …………………………………………………75
  - No bristles on epandrium are obviously feathered …………………………………………………… 79
75. At most, only one bristle on left side of epandrium is longer than those at rear margin of abdominal tergite 6……………………………………………………………………… *Megaselia hirticaudata*
  - At least two (usually more) bristles on left side of epandrium are clearly longer than those at rear margin of abdominal tergite 6 ……………………………………………………………………………… 76
76. Hairs of venter at least twice as numerous, thus segment 4 bears about 20 hairs……………………………………………………………………………… *Megaselia halterata*
  - Hairs of venter at most half as numerous, thus segment 4 bears 10 or fewer hairs …………………. 77
77. Haltere knob pale yellowish ………………………………………………………. *Megaselia Ledzona*
  - Haltere knob brown …………………………………………………………………………………… 78
78. Hind femora straw yellow …………………………………………………… *Megaselia subfuscipes*
  - Hind femora brown ………………………………………………………… *Megaselia shabestarensis*
79. Stronger, bristle-like, hairs on epandrium restricted to postero-lateral corners, there being l-2 such hairs each side………………………………………………………………… *Megaselia hendersoni*
  - The hairs of postero-ventral corners of epandrium are weaker than those on sides in front of these hair…………………………………………………………………………… *Megaselia pallidizona*
80. Knob of haltere brown …………………………………………………………………………… 81
  - Knob of haltere yellowish ………………………………………………………………………… 84
81. Terminal hairs of proctiger distinctly a little, to conspicuously, more robust than strongest hairs of cerci……………………………………………………………………… *Megaselia kermanshahensis*
  - Terminal hairs of proctiger at most only indistinctly more robust than strongest hairs on cerci, usually weaker or subequal in thickness …………………………………………………………………… 82
82. All femora dominantly yellowish, apart from dark tip to hind femur.……… *Megaselia khaghaniniai*
  - All femora somewhat pigmented, ranging from yellowish grey to blackish brown ………………… 83
83. Abdominal venter with hairs restricted to segments 5 and 6 ………………… *Megaselia propinqua*
  - Abdominal venter with hairs present on segments 3–6 ……………………… *Megaselia subnudipennis*
84. Notopleuron with only two strong bristles ……………………………………………………… 85
  - Notopleuron with three strong bristles ……………………………………………………………… 98
85. Vein Sc reaches R1, although last quarter may be a little faint …………………………………… 86
  - Vein Sc clearly ending before reaching R1 ……………………………………………………… 88
86. Terminal hairs of proctiger a little, but distinctly, more robust than hairs of cerci; a short bristle between the two strong bristles on notopleuron (even when quite short it is still longer and more robust than adjacent hairs of dorsum) …………………………………………… *Megaselia largifrontalis*
  - Terminal hairs of proctiger at most as robust as those on cerci. Any hairs between two strong bristles on notopleuron are no stronger than adjacent hairs on dorsum ………………………………………… 87
87. Hairs below basal half of hind femur longer than those of antero-ventral row of outer half………………………………………………………………… .………. *Megaselia caveonectergata*
  - Hairs below basal half of hind femur shorter than those of antero-ventral row of outer half……………………………………………………………………………. *Megaselia flavucrurus*
88. Wing membrane distinctly tinged brownish grey ………………………………………………… 89
  - Wing membrane only faintly tinged with grey ……………………………………………………… 90
89. Costa only about one-third of wing length and costal cilia relatively short ……… *Megaselia brevior*
  - Costa at least two-fifths of wing length and costal cilia longer ……………………… *Megaselia minuta*
90. A notopleural cleft present above and in front of the anterior notopleural bristle……………………………………………………………………… *Megaselia brevicostalis*
  - No notopleural cleft present …………………………………………………………………………… 91
91. Terminal hairs of proctiger at least a little, but distinctly, mote robust than hairs of cerci …………. 92
  - Terminal hairs of proctiger at most as robust as hairs on cerci ………………………………………… 93
92. Lower faces of labella with dense fields of short, pale spines …………………. *Megaselia berndseni*
  - Lower faces of labella usually with only sparsely scattered spines ……………… *Megaselia oxybelorum*
93. Pre-ocellar bristles clearly closer together than upper supra-antennals, and lower supra-antennals also well separated ……………………………………………………………………. *Megaselia perdistans*
  - Pre-ocellars as far apart or further apart than upper supra-antennals, and lower supra-antennals even closer together than latter ……………………………………………………………………………. 94
94. Lower faces of labella with few or no short pale spines …………………………….…………… 95
  - Lower faces of labella with dense fields of short, pale spine ………………………………………… 96
95. Legs yellow ………………………………………………………………………. *Megaselia haddadi*
  - Legs brown to brownish yellow …………………………………………………… *Megaselia angustiata*
96. With three bristles on axillary ridge of wing …………………………… *Megaselia miandoabensis*
  - Only two bristles on axillary ridge of wing …………………………………………………………… 97
97. Costal index less than 0.4 ……………………………………………………… *Megaselia paluventer*
  - Costal index more than 0.4 ……………………………………………………… *Megaselia barzegarae*
98. Anal tube very long relative to the length of epandrium ………………………… *Megaselia minor*
  - Anal tube shorter than the length of epandrium ………………………………………………… 99
99. Hairs of left side of epandrium only about as strong as those on cerci ……………………………. 100
  - Hairs of left side of epandrium weaker than those on cerci …………………………………………. 101
100. Lower faces of labella with numerous short pale spines …………………………… *Megaselia verna*
  - Lower faces of labella at most with a few scattered spines ………………………… *Megaselia angelicae*
101. Posterior lobe of left side of hypandrium bare on lower face ………………… *Megaselia coaetanea*
  - Posterior lobe of left side of hypandrium with fine, usually pale, hairs on lower face ………………. 102
102. All legs dominantly brown or greyish brown …………………………………………………… 103
  - At least front legs extensively yellowish …………………………………………………………… 104
103. Postero-ventral extremity of left side of epandrium more drawn out behind ……………………………………………………………………………………. *Megaselia fereagarici*
  - Postero-ventral extremity of left side of epandrium less drawn out behind ………… *Megaselia bovista*
104. Terminal hairs of proctiger only about as strong as hairs on cerci ………… *Megaselia albicaudata*
  - Terminal hairs of proctiger distinctly stronger than hairs of cerci ……………………………………105
105. Front basitarsus ventrally with hairs replaced by minute spinules ……………… *Megaselia tarsalis*
  - Front basitarsus ventrally without such a hairs ………………………………… *Megaselia farshbafi*

## Discussion

The phorid flies are very diverse in terms of species number and lifestyle but are poorly known. During this study, the phorids captured from three northwestern provinces of Iran were investigated via morphological and molecular methods, and subsequently, a genus/species-level morphological identification key was developed for male flies reported throughout the country. By comparing the known world phorid genera maintained in UCMZ, we proposed a new monotypic genus of scuttle flies, *Mahabadphora aesthesphora* gen. nov., sp. nov.

The faunistic findings revealed the presence of 13,903 males and 8,097 females during this project. All male (and some female) flies were morphologicaly identified and organized into 11 genera. *Megaselia* species (n = 13768), made up about 99% of the specimens studied (Table 2). In bulk collections of other studies, the genus *Megaselia* constitutes the most frequently captured flies [70,112–116].

The specimens of the present study were gathered from relatively restricted localities in the mountainous cold areas. Hence, with the expansion of sampling to the areas with temperate and tropical climates, we can anticipate the precise reflections of the phorid’s distribution since they are very responsive to microclimatic/habitat alterations [72,117,118].

We were able to include only 71 paratypes in our molecular experiments as the type specimens were archived in UCMZ and ICHMM collections after identity verifications. The mithocondorial *COI* and nuclear *28S*/*AK* markers were preferred to other targets because they have been proved to be informative for species-level and genus-level analyses, as indicated in a large number of resources regarding evolutionary associations in insects [e.g., 37–40,44,46,119,120].

Based on the single gene datasets, the preliminary molecular analysis of this study resulted in trees with less resolution and support; owing to fewer included characters (data are not shown). Mitochondrial markers are also more variable than nuclear ones. The mitochondrial genes help to solve the more recent divergences and nuclear ones better resolver deeper divergences, hence, the combinations of *COI*-*28S* and *COI*-*AK* datasets were applied to describe the studied paratypes, as well as to determine their relationships with known taxa. The reason for using combined analyses is that they may reliably resolve disagreements between the individual genes analyzes, enhance phylogenetic resolutions, and be more consistent with morphological data [121]. We also tried to include sequences from the same specimens in combined analyses whenever possible.

Excluding two pairs of *Megaselia* species, *M*. *hirticaudata* / *M*. *shabestarensis* and *M*. *khoyensis* / *M*. *ardabilensis*, our results specified that morphologically delimited species were congruent with the molecular analysis inferred from the *COI*-*28S* and *COI*-*AK* sequences with genetic distances and phylogenetic trees. Broadly speaking, the failure of the target genes to discriminate above-mentioned pair species is controversial. Although, belonging to the *M*. *sulphuripes* species group, *M*. *hirticaudata* and *M*. *shabestarensis* are morphologically distinct. This dissimilarity is also true for the species of *M*. *ardabilensis* and *M*. *khoyensis*. The discrepancy in morphological and molecular analysis could be a consequence of conspecificity, misidentification, or inefficiency of target genes in differentiating these species. However, the original data and photographs in combination with the quality of the sequences were carefully examined, and none of the aforesaid matters were resolved. Molecular investigations were repeated even in the case where syntypes were available, though the results did not change. According to the literature, even when two *COI* sequences are the same, there is still a chance that they belong to different taxa [122]. Therefore, to solve inconsistency like this, we suggest using supplementary loci or sequencing of the mitochondrial / nuclear genomes if possible.

Phylogenetic relationships of the understudy sequences were first examined using the NJ (S1–S3 Figs) and then by the ML method (Figs 4–6), but the second one showed more agreement with the morphological classifications. The fact that ML or Bayesian methods are more efficient than the NJ method in obtaining the true tree has been indicated in other studies [123,124].

Herein, the results of the relationship analysis were offered through three ML phylogenetic trees; the first and second trees with relying on *COI*-*28S* datasets for the *Megaselia* spp. and non*-Megaselia* species, respectively and the third tree, in support of the second one, using *COI*-*AK* sequences, to confirm the position of the newly described species within Phorinae. We reported six major clades for *Megaselia* species with low bootstrap values. Low bootstrap values may indicate that there are conflicting or little signals in the data set. Most genera within the Phoridae were monophyletic taxa with relatively a few species; however, *Megaselia* with remarkable radiation comprised of about 1,700 described species, presumably accounting for the largest genus in the animal kingdom [46,125]. Initially, the genus *Megaselia* was morphologically divided into two *Megaselia* and *Aphiochaeta* subgenera, and subsequently into further divisions and series [103,126–129]. Later, a new species (the lucifrons) group in *Megaselia* was introduced, using two *COI* and *28S* molecular markers [46]. Recently, 22 informal species groups have been proposed for this species-rich genus based on nuclear (*28S rDNA*) and mitochondrial (*ND1*, *COI*, and *16S*) markers [130]. The topology obtained for *Megaselia* sequences in this study, in agreement with other studies, represents a monophyletic lineage for this challenging genus [46,130]. Genome-scale phylogenetics is necessary to infer true monophyly and radiation of *Megaselia* species.

The last consensus tree, in support of the second one, verified *Mahabadphora aesthesphora* gen. nov., sp. nov. as a valid new taxon in the Phorinae subfamily. Both morphological and molecular analyses specified *M*. *aesthesphora* gen. nov., sp. nov. as sister taxon to *Phora* spp. Two specimens of this species were collected from West Azerbaijan, Mahabad City, which the first specimen was deposited in the UCMZ, and the second one was used for molecular analysis. This species may have a wider distribution in Iran and other areas with this type of habitat, which requires further sampling.

Literature review revealed that the phorids fauna in 12 provinces of Iran comprises of three subfamilies, 13 genera, and 97 species (Table 2). However, information on other species in the remaining 19 provinces is largely unavailable. Among 87 species offered during the current project, two new genera (*Mahabadphora* g. nov. and *Iranphora* Namaki-Khameneh & Disney, 2021) and 32 species represented new records for the world, and four genera (*Conicera*, *Dohrniphora*, *Gymnophora*, and *Triphleba*) and 47 species were new reports from Iran. Moreover, 10 species of the current study have previously been reported [49,65,69,79,100].

As a most evolutionarily successful group of macro-organisms, true flies (Diptera) can exploit almost all terrestrial and aquatic ecosystems on the earth. Indeed, Diptera is divided into families with regard to the habits (nutrition) and habitats (environment) of adults and larvae [131]. In this respect, phorid flies display the greatest diversity among all the dipterous families. The life histories of most scuttle flies are rarely documented in Iran, and limited studies have focused on only renowned species that act as the pest of edible mushrooms [49,50], invade honey bee colonies [51], or cause myiasis in humans [24]. A synopsis of bio- ecological information of 97 phorid species reported in this study was assembled from various sources and is shown in Table 2. Due to the fact that the way of life of most species is unknown, this information could expand our knowledge on the bionomics of scuttle flies in terms of environmental, agricultural, medical, and forensic prospectives.

## Conclusion

The present study is the most extensive sampling of Phoridae in Iran and the first study that utilizes the molecular characters for the identification of specimens to address morphological identification problems. Obviously, our research work has limitations in terms of sample size and sampling locations. Despite these downsides, we believe our results can comprehensively determine the taxonomic status of scuttle flies in Iran, scrutinize their phylogenetic structures, facilitate their identification and introduce a new monotypic genus.

## Supporting information

S1 Fig Neighbour joining tree inferred from 1200–1269 bp of the *COI*-*28S* gene sequences of 61 *Megaselia* species obtained in this study, along with the sequences of *M*. *scalaris* from the Genbank (KF974742-KC177721). Only bootstrap values higher than 50% are shown on the branches. The bar indicates substitutions per site. The *Drosophila melanogaster* (Meigen, 1830) (KY559392-NR_133562), *Glossina morsitans* Westwood, 1851 (KC192971**-**KC177834) and *Musca domestica* Linnaeus, 1758 (AB479529**-**AJ551427) were set as outgroups.
(TIF)

S2 Fig Neighbour joining tree inferred from 1200–1269 bp of the *COI*-*28S* gene sequences of 11 non-*Megaselia* species obtained in this study. Only bootstrap values higher than 50% are shown on the branches. The bar indicates substitutions per site. The *Drosophila melanogaster* (Meigen, 1830) (KY559392-NR_133562), *Glossina morsitans* Westwood, 1851 (KC192971**-**KC177834) and *Musca domestica* Linnaeus, 1758 (AB479529**-**AJ551427) were set as outgroups.
(TIF)

S3 Fig Neighbour joining tree inferred from 1263 bp of the *COI*-*AK* gene sequences of the new genus described in this study. Only bootstrap values higher than 50% are shown on the branches. The bar indicates substitutions per site. The *Drosophila melanogaster* (Meigen, 1830) (KY559392-U26939), *Glossina morsitans* Westwood, 1851 (KC192971**-**EZ423387) and *Musca domestica* Linnaeus, 1758 (AB479529**-**JX428899) were set as outgroups.
(TIF)

S1 Graphical Abstract (TIF)

S1 Table Inventory of sequences used in molecular analysis of the specimens of this study. I. sequences generated in this study (n = 143) which are shown in bold, II. Those used to study of interspecific and inter/intrageneric genetic diversity (n = 219), and III. Sequences applied in phylogenetic tree reconstructions (n = 204).
(DOCX)

S2 Table The LSIDs of the all publications and species mentioned in the present project. (DOCX)

S3 Table Pairwise genetic distances (%) between 71 species of Phorid species from Iran based on *COI* (down) and *28S rRNA* (up) sequences. (DOCX)

S4 Table Pairwise genetic distances of *Mahabadphora aesthesphora* gen. nov., sp. nov. from other Phorid species based on *Arginine kinase* sequences. (DOCX)
