# Supplementary figures and images for: The scuttle flies (Diptera: Phoridae) of Iran with the description of Mahabadphora aesthesphora as a new genus and species
Source: PLoS One. 2021 Oct 13;16(10):e0257899. doi: 10.1371/journal.pone.0257899 (PMC8513852; doi:10.1371/journal.pone.0257899)

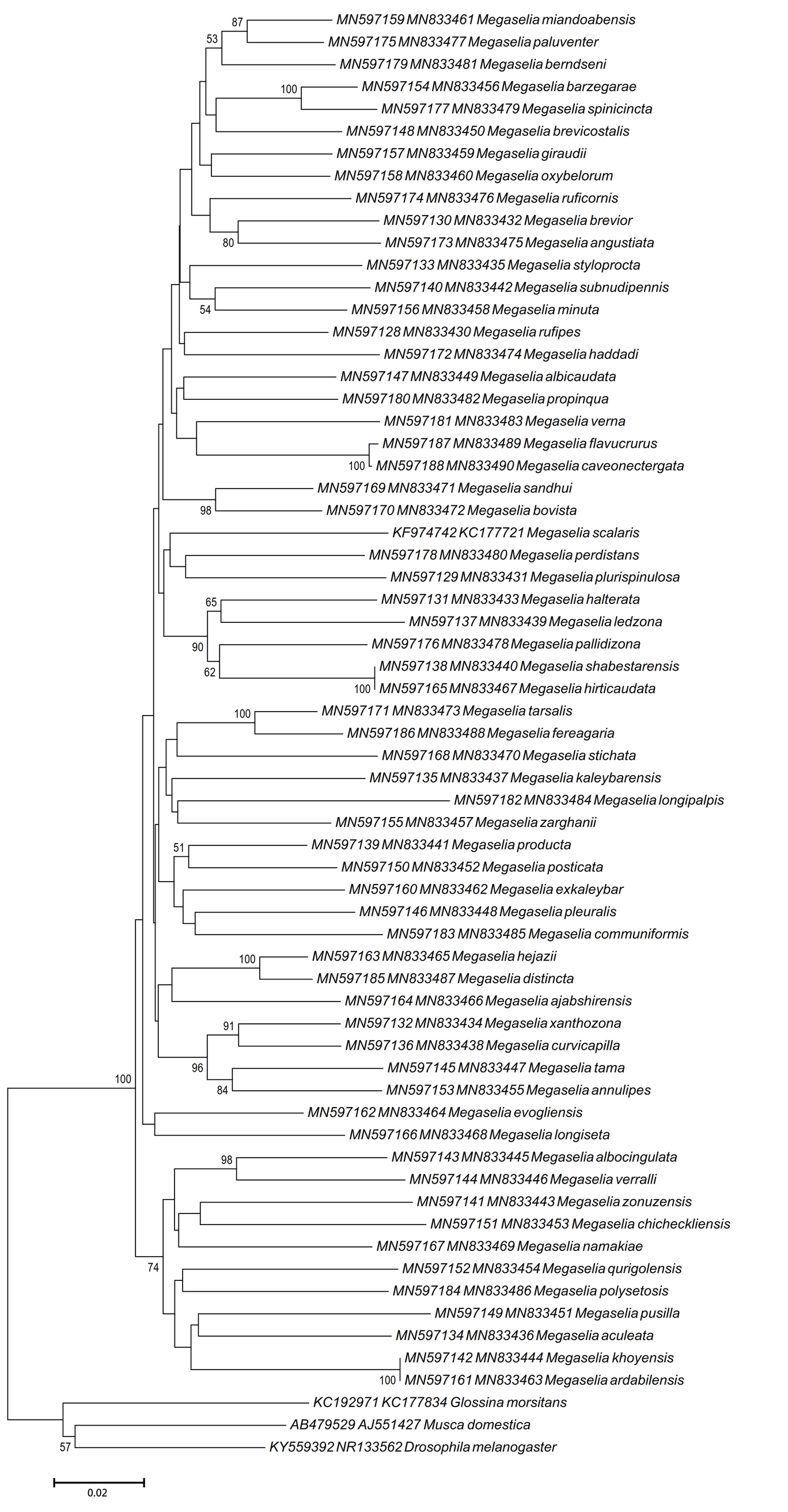

Supplement: S1 Fig — Only bootstrap values higher than 50% are shown on the branches. The bar indicates substitutions per site. The Drosophila melanogaster (Meigen, 1830) (KY559392-NR_133562), Glossina morsitans Westwood, 1851 (KC192971-KC177834) and Musca domestica Linnaeus, 1758 (AB479529-AJ551427) were set as outgroups. (TIF) [file pone.0257899.s001.tif]

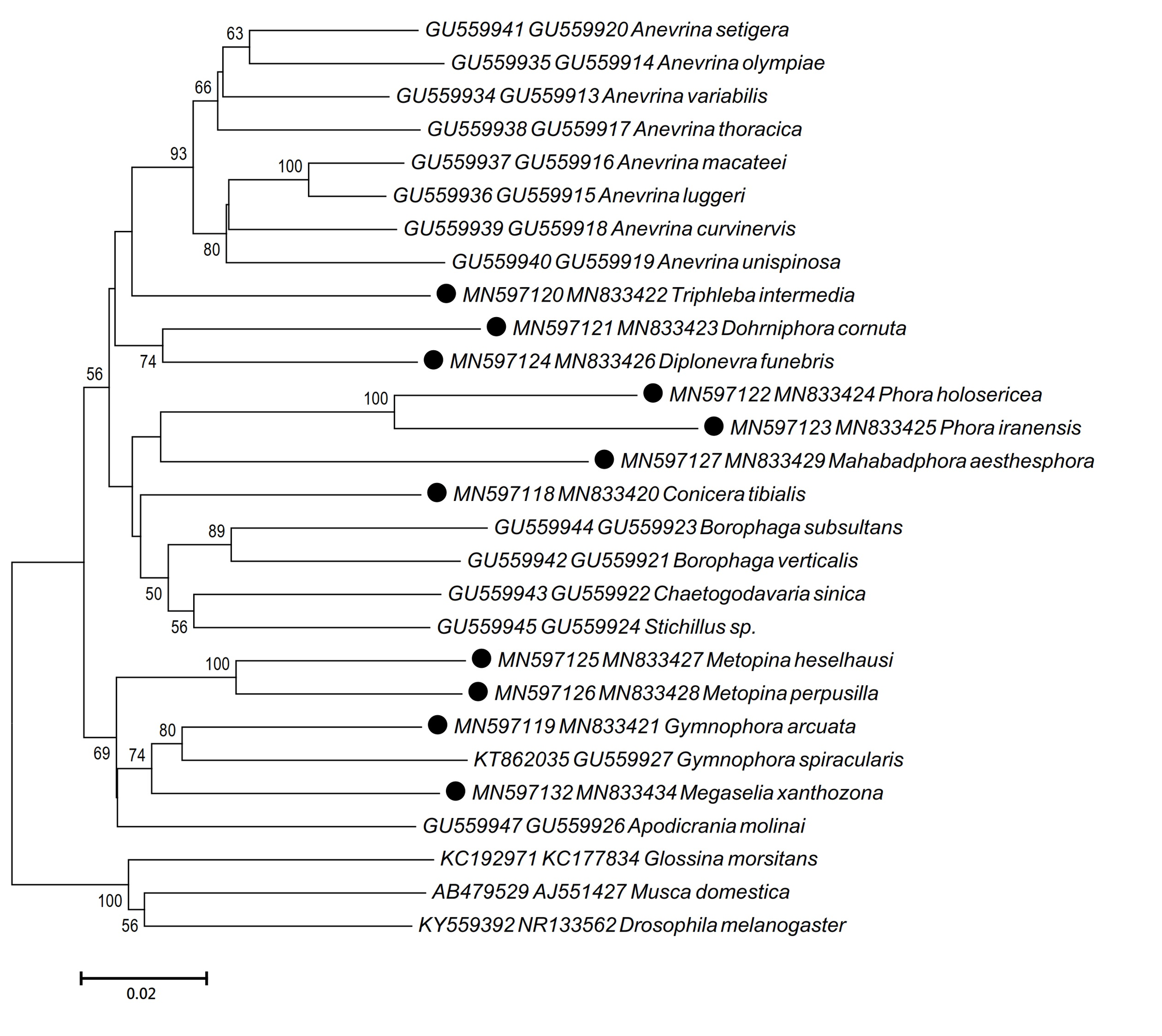

Supplement: S2 Fig — Only bootstrap values higher than 50% are shown on the branches. The bar indicates substitutions per site. The Drosophila melanogaster (Meigen, 1830) (KY559392-NR_133562), Glossina morsitans Westwood, 1851 (KC192971-KC177834) and Musca domestica Linnaeus, 1758 (AB479529-AJ551427) were set as outgroups. (TIF) [file pone.0257899.s002.tif]

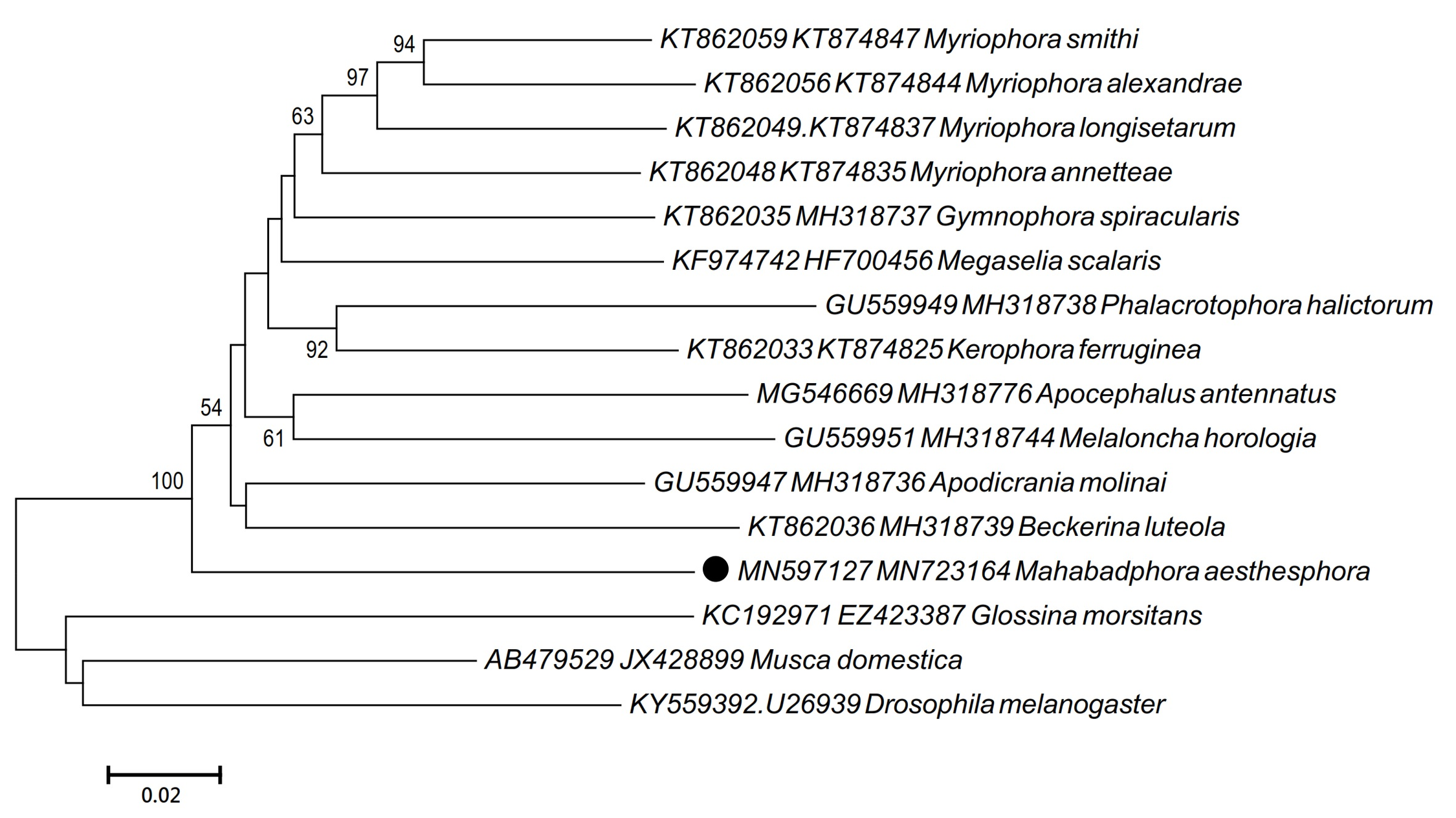

Supplement: S3 Fig — Only bootstrap values higher than 50% are shown on the branches. The bar indicates substitutions per site. The Drosophila melanogaster (Meigen, 1830) (KY559392-U26939), Glossina morsitans Westwood, 1851 (KC192971-EZ423387) and Musca domestica Linnaeus, 1758 (AB479529-JX428899) were set as outgroups. (TIF) [file pone.0257899.s003.tif]

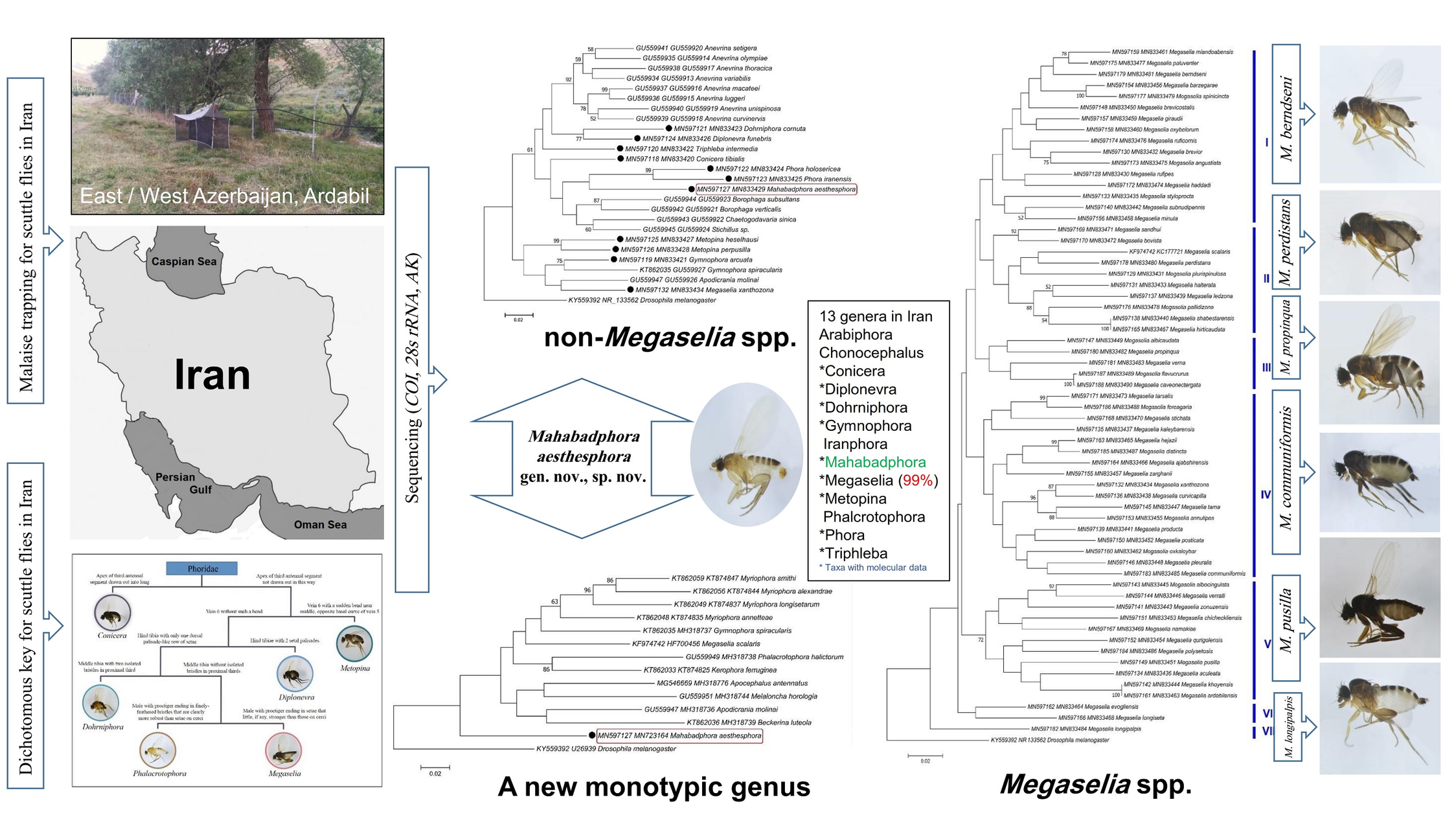

Supplement: S1 Graphical Abstract — (TIF) [file pone.0257899.s004.tif]
