# Supplementary material for: The scuttle flies (Diptera: Phoridae) of Iran with the description of Mahabadphora aesthesphora as a new genus and species
Source: PLoS One. 2021 Oct 13;16(10):e0257899. doi: 10.1371/journal.pone.0257899 (PMC8513852; doi:10.1371/journal.pone.0257899)
Supplement: S1 Table — I. sequences generated in this study (n = 143) which are shown in bold, II. Those used to study of interspecific and inter/intrageneric genetic diversity (n = 219), and III. Sequences applied in phylogenetic tree reconstructions (n = 204). (DOCX) [file pone.0257899.s005.docx]

**Supplementary Table 1**. Inventory of sequences used in molecular analysis of the specimens of this study; I. sequences generated in this study (n=143) which are shown in bold, II. Those used to study of interspecific and inter/intrageneric genetic diversity (n=219), and III. Sequences applied in phylogenetic tree reconstructions (n=204).

| **No.** | **Species** | **GeneBank Accession numbers** | | | **Sequences** | | | | | |
| --- | --- | --- | --- | --- | --- | --- | --- | --- | --- | --- |
|  |  |  |  |  | **Genetic distances** | | | **Phylogenetic study** | | |
|  |  | ***COI*** | ***28S*** | ***AK*** | ***COI*** | ***28S*** | ***AK*** | ***COI*-*28S* dataset for analysis of *Megaselia* spp.** | ***COI*-*28S* dataset for analysis of**  **non-*Megaselia* spp.** | ***COI-AK* dataset for analysis of the new genus/species** |
|  | **Outgroups** | | | | | | | | | |
| 1 | *Glossina.morsitans* | KC192971 | KC177834 | EZ423387 |  |  |  | * | * | * |
| 2 | *Drosophila.melanogaster* | KY559392 | NR_133562 | U26939 |  |  |  | * | * | * |
| 3 | *Musca.domestica* | AB479529 | AJ551427 | JX428899 |  |  |  | * | * | * |
|  | **Metopininae** | | | | | | | | | |
| 4 | *Apocephalus antennatus* | MG546669 |  | MH318776 | * |  | * |  |  | * |
| 5 | *Apocephalus analis* |  |  | MH318747 |  |  | * |  |  |  |
| 6 | *Apodicrania molinai* | GU559947 | GU559926 | MH318736 | * | * | * |  | * | * |
| 7 | *Beckerina luteola* | KT862036 |  | MH318739 | * |  | * |  |  | * |
| **8** | ***Gymnophora arcuata*** | **MN597119** | **MN833421** |  | * | * |  |  | * |  |
| 9 | *Gymnophora spiracularis* | KT862035 | GU559927 | MH318737 | * | * | * |  | * | * |
| 10 | *Kerophora.ferruginea* | KT862033 |  | KT874825 |  |  | * |  |  | * |
| **11** | ***Megaselia albicaudata*** | **MN597147** | **MN833449** |  | * | * |  | * |  |  |
| **12** | ***Megaselia aculeata*** | **MN597134** | **MN833436** |  | * | * |  | * |  |  |
| **13** | ***Megaselia albocingulata*** | **MN597143** | **MN833445** |  | * | * |  | * |  |  |
| **14** | ***Megaselia ajabshirensis*** | **MN597164** | **MN833466** |  | * | * |  | * |  |  |
| **15** | ***Megaselia angustiata*** | **MN597173** | **MN833475** |  | * | * |  | * |  |  |
| **16** | ***Megaselia ardabilensis*** | **MN597161** | **MN833463** |  | * | * |  | * |  |  |
| **17** | ***Megaselia annulipes*** | **MN597153** | **MN833455** |  | * | * |  | * |  |  |
| **18** | ***Megaselia barzegarae*** | **MN597154** | **MN833456** |  | * | * |  | * |  |  |
| **19** | ***Megaselia brevicostalis*** | **MN597148** | **MN833450** |  | * | * |  | * |  |  |
| **20** | ***Megaselia brevior*** | **MN597130** | **MN833432** |  | * | * |  | * |  |  |
| **21** | ***Megaselia bovista*** | **MN597170** | **MN833472** |  | * | * |  | * |  |  |
| **22** | ***Megaselia berndseni*** | **MN597179** | **MN833481** |  | * | * |  | * |  |  |
| **23** | ***Megaselia curvicapilla*** | **MN597136** | **MN833438** |  | * | * |  | * |  |  |
| **24** | ***Megaselia chicheckliensis*** | **MN597151** | **MN833453** |  | * | * |  | * |  |  |
| **25** | ***Megaselia caveonectergata*** | **MN597188** | **MN833490** |  | * | * |  | * |  |  |
| **26** | ***Megaselia communiformis*** | **MN597183** | **MN833485** |  | * | * |  | * |  |  |
| **27** | ***Megaselia distincta*** | **MN597185** | **MN833487** |  | * | * |  | * |  |  |
| **28** | ***Megaselia exkaleybar*** | **MN597160** | **MN833462** |  | * | * |  | * |  |  |
| **29** | ***Megaselia evogliensis*** | **MN597162** | **MN833464** |  | * | * |  | * |  |  |
| **30** | ***Megaselia fereagarici*** | **MN597186** | **MN833488** |  | * | * |  | * |  |  |
| **31** | ***Megaselia flavucrurus*** | **MN597187** | **MN833489** |  | * | * |  | * |  |  |
| **32** | ***Megaselia giraudii*** | **MN597157** | **MN833459** |  | * | * |  | * |  |  |
| **33** | ***Megaselia haddadi*** | **MN597172** | **MN833474** |  | * | * |  | * |  |  |
| **34** | ***Megaselia halterata*** | **MN597131** | **MN833433** |  | * | * |  | * |  |  |
| **35** | ***Megaselia hejazii*** | **MN597163** | **MN833465** |  | * | * |  | * |  |  |
| **36** | ***Megaselia hirticaudata*** | **MN597165** | **MN833467** |  | * | * |  | * |  |  |
| **37** | ***Megaselia khoyensis*** | **MN597142** | **MN833444** |  | * | * |  | * |  |  |
| **38** | ***Megaselia kaleybarensis*** | **MN597135** | **MN833437** |  | * | * |  | * |  |  |
| **39** | ***Megaselia longiseta*** | **MN597166** | **MN833468** |  | * | * |  | * |  |  |
| **40** | ***Megaselia ledzona*** | **MN597137** | **MN833439** |  | * | * |  | * |  |  |
| **41** | ***Megaselia longipalpis*** | **MN597182** | **MN833484** |  | * | * |  | * |  |  |
| **42** | ***Megaselia minuta*** | **MN597156** | **MN833458** |  | * | * |  | * |  |  |
| **43** | ***Megaselia miandoabensis*** | **MN597159** | **MN833461** |  | * | * |  | * |  |  |
| 44 | *Megaselia mithridatesi* | KT862044 |  |  | * |  |  |  |  |  |
| **45** | ***Megaselia namakiae*** | **MN597167** | **MN833469** |  | * | * |  | * |  |  |
| **46** | ***Megaselia oxybelorum*** | **MN597158** | **MN833460** |  | * | * |  | * |  |  |
| **47** | ***Megaselia plurispinulosa*** | **MN597129** | **MN833431** |  | * | * |  | * |  |  |
| **48** | ***Megaselia producta*** | **MN597139** | **MN833441** |  | * | * |  | * |  |  |
| **49** | ***Megaselia pleuralis*** | **MN597146** | **MN833448** |  | * | * |  | * |  |  |
| **50** | ***Megaselia propinqua*** | **MN597180** | **MN833482** |  | * | * |  | * |  |  |
| **51** | ***Megaselia pusilla*** | **MN597149** | **MN833451** |  | * | * |  | * |  |  |
| **52** | ***Megaselia posticata*** | **MN597150** | **MN833452** |  | * | * |  | * |  |  |
| **53** | ***Megaselia perdistans*** | **MN597178** | **MN833480** |  | * | * |  | * |  |  |
| **54** | ***Megaselia polysetosis*** | **MN597184** | **MN833486** |  | * | * |  | * |  |  |
| **55** | ***Megaselia paluventer*** | **MN597175** | **MN833477** |  | * | * |  | * |  |  |
| **56** | ***Megaselia pallidizona*** | **MN597176** | **MN833478** |  | * | * |  | * |  |  |
| **57** | ***Megaselia qurigolensis*** | **MN597152** | **MN833454** |  | * | * |  | * |  |  |
| **58** | ***Megaselia ruficornis*** | **MN597174** | **MN833476** |  | * | * |  | * |  |  |
| **59** | ***Megaselia rufipes*** | **MN597128** | **MN833430** |  | * | * |  | * |  |  |
| **60** | ***Megaselia styloprocta*** | **MN597133** | **MN833435** |  | * | * |  | * |  |  |
| **61** | ***Megaselia shabestarensis*** | **MN597138** | **MN833440** |  | * | * |  | * |  |  |
| **62** | ***Megaselia subnudipennis*** | **MN597140** | **MN833442** |  | * | * |  | * |  |  |
| 63 | *Megaselia scalaris* | KF974742 | KC177721 | HF700456 | * | * | * | * |  | * |
| 64 | *Megaselia scalaris* | KC192982 |  |  | * |  |  |  |  |  |
| **65** | ***Megaselia stichata*** | **MN597168** | **MN833470** |  | * | * |  | * |  |  |
| **66** | ***Megaselia sandhui*** | **MN597169** | **MN833471** |  | * | * |  | * |  |  |
| **67** | ***Megaselia spinicincta*** | **MN597177** | **MN833479** |  | * | * |  | * |  |  |
| 68 | *Megaselia spiracularis* | MN832848 |  |  | * |  |  |  |  |  |
| **69** | ***Megaselia tama*** | **MN597145** | **MN833447** |  | * | * |  | * |  |  |
| **70** | ***Megaselia tarsalis*** | **MN597171** | **MN833473** |  | * | * |  | * |  |  |
| **71** | ***Megaselia verna*** | **MN597181** | **MN833483** |  | * | * |  | * |  |  |
| **72** | ***Megaselia verralli*** | **MN597144** | **MN833446** |  | * | * |  | * |  |  |
| **73** | ***Megaselia xanthozona*** | **MN597132** | **MN833434** |  | * | * |  | * | * |  |
| **74** | ***Megaselia zarghanii*** | **MN597155** | **MN833457** |  | * | * |  | * |  |  |
| **75** | ***Megaselia zonuzensis*** | **MN597141** | **MN833443** |  | * | * |  | * |  |  |
| 76 | *Melaloncha horologia* | GU559951 |  | MH318744 | * |  | * |  |  | * |
| **77** | ***Metopina heselhausi*** | **MN597125** | **MN833427** |  | * | * |  |  | * |  |
| **78** | ***Metopina perpusilla*** | **MN597126** | **MN833428** |  | * | * |  |  | * |  |
| 79 | *Myriophora alexandrae* | KT862056 |  | KT874844 | * |  | * |  |  | * |
| 80 | *Myriophora annetteae* | KT862048 |  | KT874835 | * |  | * |  |  | * |
| 81 | *Myriophora angustifascia* | KT862047 |  |  | * |  |  |  |  |  |
| 82 | *Myriophora communis* | KT862045 |  |  | * |  |  |  |  |  |
| 83 | *Myriophora smithi* | KT862059 |  | KT874847 |  |  | * |  |  | * |
| 84 | *Myriophora.longisetarum* | KT862049 |  | KT874837 |  |  | * |  |  | * |
| 85 | *Phalacrotophora halictorum* | GU559949 |  | MH318738 | * |  | * |  |  | * |
|  | **Phorinae** | | | | | | | | | |
| 86 | *Anevrina curvinervis* | GU559939 | GU559918 |  | * | * |  |  | * |  |
| 87 | *Anevrina macateei* | GU559937 | GU559916 |  | * | * |  |  | * |  |
| 88 | *Anevrina setigera* | GU559941 | GU559920 |  | * | * |  |  | * |  |
| 89 | *Anevrina thoracica* | GU559938 | GU559917 |  | * | * |  |  | * |  |
| 90 | *Anevrina unispinosa* | GU559940 | GU559919 |  | * | * |  |  | * |  |
| 91 | *Anevrina variabilis* | GU559934 | GU559913 |  | * | * |  |  | * |  |
| 92 | *Anevrina luggeri* | GU559936 | GU559915 |  | * | * |  |  | * |  |
| 93 | *Anevrina olympiae* | GU559935 | GU559914 |  | * | * |  |  | * |  |
| 94 | *Borophaga subsultans* | GU559944 | GU559923 |  | * | * |  |  | * |  |
| 95 | *Borophaga verticalis* | GU559942 | GU559921 |  | * | * |  |  | * |  |
| 96 | *Chaetogodavaria sinica* | GU559943 | GU559922 |  | * | * |  |  | * |  |
| 97 | *Chaetopleurophora asiatica* | KF601894 |  |  | * |  |  |  |  |  |
| **98** | ***Conicera tibialis*** | **MN597118** | **MN833420** |  | * | * |  |  | * |  |
| 99 | *Conicera dauci* |  | JN664719 |  |  | * |  |  |  |  |
| 100 | *Conicera dauci* |  | HM062616 |  |  | * |  |  |  |  |
| 101 | *Diplonevra bifasciata* | HM352591 |  |  | * |  |  |  |  |  |
| **102** | ***Diplonevra funebris*** | **MN597124** | **MN833426** |  | * | * |  |  | * |  |
| 103 | *Diplonevra funebris* | HM352585 |  |  | * |  |  |  |  |  |
| 104 | *Diplonevra hamata* | HM352589 |  |  | * |  |  |  |  |  |
| 105 | *Diplonevra gaudialis* | HM352608 |  |  | * |  |  |  |  |  |
| 106 | *Diplonevra sp.* | HM352560 |  |  | * |  |  |  |  |  |
| 107 | *Dohrniphora berezovskiyi* | HM352573 |  |  | * |  |  |  |  |  |
| 108 | *Dohrniphora buscki* | HM352569 |  |  | * |  |  |  |  |  |
| 109 | *Dohrniphora circumflexa* | HM352578 |  |  | * |  |  |  |  |  |
| **110** | ***Dohrniphora cornuta*** | **MN597121** | **MN833423** |  | * | * |  |  | * |  |
| 111 | *Dohrniphora cornuta* | MN832849 |  |  | * |  |  |  |  |  |
| 112 | *Dohrniphora divaricate* | HM352584 |  |  | * |  |  |  |  |  |
| 113 | *Dohrniphora ecitophila* | HM352570 |  |  | * |  |  |  |  |  |
| 114 | *Dohrniphora ecitophila* | HM352564 |  |  | * |  |  |  |  |  |
| 115 | *Dohrniphora membranea* | HM352580 |  |  | * |  |  |  |  |  |
| 116 | *Dohrniphora porrasae* | HM352604 |  |  | * |  |  |  |  |  |
| 117 | *Dohrniphora porrasae* | HM352561 |  |  | * |  |  |  |  |  |
| 118 | *Dohrniphora procera* | HM352614 |  |  | * |  |  |  |  |  |
| 119 | *Dohrniphora segregata* | HM352619 |  |  | * |  |  |  |  |  |
| **120** | ***Phora holosericea*** | **MN597122** | **MN833424** |  | * | * |  |  | * |  |
| **121** | ***Phora iranensis*** | **MN597123** | **MN833425** |  | * | * |  |  | * |  |
| 122 | *Latiborophaga sp.* | HM352620 |  |  | * |  |  |  |  |  |
| **123** | ***Mahabadphora aesthesphora*** | **MN597127** | **MN833429** | **MN723164** | * | * | * |  | * | * |
| 124 | *Stichillus sp.* | GU559945 | GU559924 |  | * | * |  |  | * |  |
| 125 | *Stichillus spinosus* | HM352622 |  |  | * |  |  |  |  |  |
| **126** | ***Triphleba intermedia*** | **MN597120** | **MN833422** |  | * | * |  |  | * |  |
