# Supplementary material for: The scuttle flies (Diptera: Phoridae) of Iran with the description of Mahabadphora aesthesphora as a new genus and species
Source: PLoS One. 2021 Oct 13;16(10):e0257899. doi: 10.1371/journal.pone.0257899 (PMC8513852; doi:10.1371/journal.pone.0257899)
Supplement: S2 Table — (DOCX) [file pone.0257899.s006.docx]

**Supplementary Table 2**. The LSIDs of the all publications and species mentioned in the present project

**Paper 1**: [Namaki Khameneh, Roya, Samad Khaghaninia & Ronald H. L. Disney. 2019](http://zoobank.org/References/b28c521b-2bfe-44f3-9041-1656341f29b6) Scuttle flies (Diptera: Phoridae) from Ardabil province in Iran, with description of a new species of *Megaselia* Rondani. *Zoology in the Middle East* 65(1): 70-74.

LSIDurn:lsid:zoobank.org:pub:80C3BBC8-BE63-46DC-9372-9E682B235129

Species:

***Megaselia ardabilensis*** Namaki Khameneh, Khaghaninia, Disney, 2019

LSIDurn:lsid:zoobank.org:act:639C02AD-2105-44AA-B1F9-D8C625A27B38

**Paper 2**: Namaki Khameneh, Roya, Samad Khaghaninia, R. H. L. Disney & Naseh Maleki-Ravasan. 2019. Twenty one new species of *Megaselia* Rondani (Diptera: Phoridae) from Iran. *Zootaxa* 4711(1): 1–50.

LSIDurn:lsid:zoobank.org:pub:604227AA-58EB-408C-8794-6E30192C3F74

Species:

***Megaselia ajabshirensis*** Namaki Khameneh, Khaghaninia, Disney & Maleki-Ravasan, 2019

LSIDurn:lsid:zoobank.org:act:6D111732-AA5B-4A0B-B583-6E6404E58804

***Megaselia chicheckliensis*** Namaki Khameneh, Khaghaninia, Disney & Maleki-Ravasan, 2019

LSIDurn:lsid:zoobank.org:act:71004DD9-5622-46F8-BFCF-C1F75BC31329

***Megaselia exkaleybar*** Namaki Khameneh, Khaghaninia, Disney & Maleki-Ravasan, 2019

LSIDurn:lsid:zoobank.org:act:A74764C1-8D8C-4E44-A7B1-F86103B73251

***Megaselia kaleybarensis*** Namaki Khameneh, Khaghaninia, Disney & Maleki-Ravasan, 2019

LSIDurn:lsid:zoobank.org:act:89AE9B13-F2FA-40CE-8349-71971C367F2E

***Megaselia qurigolensis*** Namaki Khameneh, Khaghaninia, Disney & Maleki-Ravasan, 2019

LSIDurn:lsid:zoobank.org:act:EEF2270A-ADBB-4D95-8370-3AA592FB06CB

***Megaselia shabestarensis*** Namaki Khameneh, Khaghaninia, Disney & Maleki-Ravasan, 2019

LSIDurn:lsid:zoobank.org:act:B01A60D8-5118-4279-B42E-9517C8EE9C97

***Megaselia zonuzensis*** Namaki Khameneh, Khaghaninia, Disney & Maleki-Ravasan, 2019

LSIDurn:lsid:zoobank.org:act:6C8F16D7-E643-4931-AC55-42071E3B5429

***Megaselia evogliensis*** Namaki Khameneh, Khaghaninia, Disney & Maleki-Ravasan, 2019

LSIDurn:lsid:zoobank.org:act:935F5A25-451A-4FC5-BFD8-11E0B24735D7

***Megaselia farshbafi*** Namaki Khameneh, Khaghaninia, Disney & Maleki-Ravasan, 2019

LSIDurn:lsid:zoobank.org:act:2C84C310-4D14-435B-ACB9-2FB02E3EFBFF

***Megaselia ghalateshahensis*** Namaki Khameneh, Khaghaninia, Disney & Maleki-Ravasan, 2019

LSIDurn:lsid:zoobank.org:act:3A0EC3DB-CBA6-43A3-877E-82D05D67F925

***Megaselia haddadi*** Namaki Khameneh, Khaghaninia, Disney & Maleki-Ravasan, 2019

LSIDurn:lsid:zoobank.org:act:8AD322C8-5C0B-49E6-92D2-CBAC70B763D4

***Megaselia hejazii*** Namaki Khameneh, Khaghaninia, Disney & Maleki-Ravasan, 2019

LSIDurn:lsid:zoobank.org:act:E39B4CA5-0FFC-4644-9DA1-6D02F496CA4B

***Megaselia khaghaniniai*** Namaki Khameneh & Disney in Namaki Khameneh, Khaghaninia, Disney & Maleki-Ravasan, 2019

LSIDurn:lsid:zoobank.org:act:DF2F5427-CE2E-4AC7-BE19-0CBD24F98514

***Megaselia khoyensis*** Namaki Khameneh, Khaghaninia, Disney & Maleki-Ravasan, 2019

LSIDurn:lsid:zoobank.org:act:6FBF7B3F-035B-4C49-868F-2D442B280D50

***Megaselia ledzona*** Namaki Khameneh, Khaghaninia, Disney & Maleki-Ravasan, 2019

LSIDurn:lsid:zoobank.org:act:C651200A-DEEC-432B-957F-FAAEBC6DBB9E

***Megaselia mahabadensis*** Namaki Khameneh, Khaghaninia, Disney & Maleki-Ravasan, 2019

LSIDurn:lsid:zoobank.org:act:D37C00A1-050F-4BFB-B75C-16E59C5D2A23

***Megaselia miandoabensis*** Namaki Khameneh, Khaghaninia, Disney & Maleki-Ravasan, 2019

LSIDurn:lsid:zoobank.org:act:A0459A3B-0969-4384-AC5B-BD2E99C84193

***Megaselia namakiae*** Khaghaninia & Disney in Namaki Khameneh, Khaghaninia, Disney & Maleki-Ravasan, 2019

LSIDurn:lsid:zoobank.org:act:05BDFC31-91F9-4F0B-B4B2-FE056ED60D97

***Megaselia pereensis*** Namaki Khameneh, Khaghaninia, Disney & Maleki-Ravasan, 2019

LSIDurn:lsid:zoobank.org:act:45A1BBBA-F680-40D0-9181-86278EB529D7

***Megaselia yaseri*** Namaki Khameneh, Khaghaninia, Disney & Maleki-Ravasan, 2019

LSIDurn:lsid:zoobank.org:act:0FF05021-5010-4BC8-81D6-D70E41507BC3

***Megaselia zarghanii*** Namaki Khameneh, Khaghaninia, Disney & Maleki-Ravasan, 2019

LSIDurn:lsid:zoobank.org:act:D262359E-9585-469E-978E-3FDAB5992FDE

**Paper 3**: Namaki-Khameneh, Roya, Samad Khaghaninia, Ronald H. L. Disney & Naseh Maleki-Ravasan. 2021. Nine new species of scuttle flies, including one new genus (Diptera: Phoridae) from Iran. *Biologia*.

LSIDurn:lsid:zoobank.org:pub:FD6A0939-EB17-415D-A4B2-B1EF0F172033

Genus:

***Iranphora*** Namaki-Khameneh & Disney in Namaki-Khameneh, Khaghaninia, Disney & Maleki-Ravasan, 2021

LSIDurn:lsid:zoobank.org:act:9A960F1D-DA56-4DDB-A37C-291E97E71E55

Species:

***Iranphora sharafkhaneensis*** Namaki-Khameneh & Disney in Namaki-Khameneh, Khaghaninia, Disney & Maleki-Ravasan, 2021

LSIDurn:lsid:zoobank.org:act:41418655-67A3-4AA4-B15B-B86BE2A59193

***Megaselia caveonectergata*** Namaki-Khameneh & Disney in Namaki-Khameneh, Khaghaninia, Disney & Maleki-Ravasan, 2021

LSIDurn:lsid:zoobank.org:act:77B05CA0-F46C-4178-958B-D1B8DC3C384D

***Megaselia distincta*** Namaki-Khameneh & Disney in Namaki-Khameneh, Khaghaninia, Disney & Maleki-Ravasan, 2021

LSIDurn:lsid:zoobank.org:act:D94288EE-7861-4388-88E9-868475615D1B

***Megaselia fereagarici*** Namaki-Khameneh & Disney in Namaki-Khameneh, Khaghaninia, Disney & Maleki-Ravasan, 2021

LSIDurn:lsid:zoobank.org:act:C6DEA213-12A5-416B-82B1-9CBB8603CD1C

***Megaselia flavucrurus*** Namaki-Khameneh & Disney in Namaki-Khameneh, Khaghaninia, Disney & Maleki-Ravasan, 2021

LSIDurn:lsid:zoobank.org:act:E21E5A64-1100-41BC-B144-DB1CE1AF7F81

***Megaselia paluventer*** Namaki-Khameneh & Disney in Namaki-Khameneh, Khaghaninia, Disney & Maleki-Ravasan, 2021

LSIDurn:lsid:zoobank.org:act:E3D25165-8251-4045-89B4-024E23A14E1E

***Megaselia polysetosis*** Namaki-Khameneh & Disney in Namaki-Khameneh, Khaghaninia, Disney & Maleki-Ravasan, 2021

LSIDurn:lsid:zoobank.org:act:803E99B0-A0ED-462B-A18C-FA51F9850F81

***Phalacrotophora flavidus*** Namaki-Khameneh & Disney in Namaki-Khameneh, Khaghaninia, Disney & Maleki-Ravasan, 2021

LSIDurn:lsid:zoobank.org:act:7AD206AF-72F8-4B2A-8372-66A9608FFFB3

***Phora iranensis*** Namaki-Khameneh & Disney in Namaki-Khameneh, Khaghaninia, Disney & Maleki-Ravasan, 2021

LSIDurn:lsid:zoobank.org:act:0C9B5005-2687-4F73-A3A0-97148B3BB3AF

**Paper 4**: Namaki-Khameneh, Roya, Samad Khaghaninia, Ronald H. L. Disney & Naseh Maleki-Ravasan. In Press. The scuttle flies (Diptera: Phoridae) of Iran with the description of *Mahabadphora aesthesphora* as a new genus and species. *PLOS ONE.*

LSIDurn:lsid:zoobank.org:pub:9145941B-10BF-4B27-8B4C-D90006A857B5

Genus:

***Mahabadphora*** Namaki-Khameneh & Disney in Namaki-Khameneh, Khaghaninia, Disney & Maleki-Ravasan, In Press

LSIDurn:lsid:zoobank.org:act:BB5DCD6D-EFA2-4E34-828A-C34A1438E093

Species:

***Mahabadphora aesthesphora*** Namaki-Khameneh & Disney in Namaki-Khameneh, Khaghaninia, Disney & Maleki-Ravasan, In Press

LSIDurn:lsid:zoobank.org:act:4FD302BB-CBAF-45E2-B2D2-D53F6DF1DCA0
