# Supplementary material for: The scuttle flies (Diptera: Phoridae) of Iran with the description of Mahabadphora aesthesphora as a new genus and species
Source: PLoS One. 2021 Oct 13;16(10):e0257899. doi: 10.1371/journal.pone.0257899 (PMC8513852; doi:10.1371/journal.pone.0257899)
Supplement: S3 Table — (DOCX) [file pone.0257899.s007.docx]

**Supplementary Table 3**. Pairwise genetic distances (%) between 71 species of Phorid species from Iran based on *COI* (down) and *28S rRNA* (up) sequences
