# Supplementary material for: The scuttle flies (Diptera: Phoridae) of Iran with the description of Mahabadphora aesthesphora as a new genus and species
Source: PLoS One. 2021 Oct 13;16(10):e0257899. doi: 10.1371/journal.pone.0257899 (PMC8513852; doi:10.1371/journal.pone.0257899)
Supplement: S4 Table — (DOCX) [file pone.0257899.s008.docx]

**Supplementary Table 4**.Pairwise genetic distances of *Mahabadphora aesthesphora* gen. nov., sp. nov. from other Phorid species based on *Arginine kinase* sequences
